# Annotated checklist of the invertebrate macrozoobenthos from Mauritanian marine shallow-water habitats

**DOI:** 10.3897/zookeys.1277.180972

**Published:** 2026-04-09

**Authors:** Alexander H. Knorrn, Moritz Sonnewald, Sidi Mohamed M. Moctar, Mamadou Dia, Ely Beibou, André Freiwald

**Affiliations:** 1 Senckenberg am Meer, Marine Research Department, Wilhelmshaven, Germany Senckenberg am Meer, Marine Research Department Wilhelmshaven Germany; 2 MARUM, University of Bremen, Bremen, Germany MARUM, University of Bremen Bremen Germany; 3 Senckenberg Research Institute and Natural History Museum, Department of Marine Zoology, Section Ichthyology, Frankfurt/M, Germany Institut Mauritanien de Recherches Océanographiques et des Pêches Nouadhibou Mauritania; 4 Institut Mauritanien de Recherches Océanographiques et des Pêches (IMROP), Nouadhibou, Mauritania Senckenberg Research Institute and Museum of Nature, Department of Marine Zoology, Section Ichthyology Frankfurt/M Germany

**Keywords:** Biodiversity, macrozoobenthos, upwelling, West Africa

## Abstract

From 2020 to 2024, The Institut Mauritanien de Recherches Océanographiques et des Pêche (IMROP) and the Senckenberg Research Institute conducted five joint expeditions, exploring the marine biodiversity of the North-Mauritanian coastal habitats, primarily focusing on the Baie de l’Étoile north of Nouadhibou and to the Banc d’Arguin National Park. In order to establish a Mauritanian scientific reference collection of the marine fauna and to build up a DNA barcode library, macrozoobenthic invertebrates from all major groups were collected using various methods and gear. A total of 103 living marine macrozoobenthic invertebrate species were found and their key morphological features were described herein. This checklist gives an overview on the most common species of the Mauritanian coastal macroinvertebrates and provides a baseline for future biodiversity assessments along the Mauritanian coast.

## Introduction

Invertebrates constitute an essential component of every marine food web. However, their ecological significance is often underestimated and overlooked in comparison to commercially exploited groups such as fish or larger molluscs (Collier et al. 2016; Eisenhauer et al. 2019; Chen 2021). Consequently, conventional single-species models have frequently led to unsustainable exploitation of fish stocks, as they fail to consider the trophic resources like marine invertebrates and their predator interactions (Perryman et al. 2021). By contrast, more sustainable and holistic approaches, such as ecosystem-based fisheries management (EBFM), integrate not only the target species but also environ­mental drivers and key prey resources, including marine invertebrates, into the stock assessments (Karp et al. 2023). Incorporating these broader ecological relationships enables an earlier detection of changes within the food web and, consequently, facilitates the anticipation of potential stock collapses. However, the successful implementation of EBFM critically depends on robust local taxonomic knowledge. This is particularly important in patchy studied marine regions, such as the Mauritanian waters (Knorrn et al. 2025a), where establishing a solid species-specific documentation of the local fauna is of great importance for a future effective fisheries management (Ullah et al. 2023).

The Mauritanian coast is situated within the Canary Current Large Marine Ecosystem (CCLME), which is subject to strong seasonal upwelling activities (Heileman and Tandstad 2009). This upwelling occurs when cold, nutrient-rich deep water is brought to the surface by offshore winds, resulting in high nutrient concentrations in the upper water layers. This nutrient surplus leads to large phytoplankton blooms, which form the basis for extensive food webs and support some of the most productive fish stocks in the Atlantic Ocean. The surplus of nutrients and the geographic location of Mauritania contribute to a remarkably high biodiversity along its coast, encompassing species from temperate, tropical, and subtropical regions (Jager 1993; Le Loeuff and von Cosel 1998; Knorrn et al. 2025b). The northernmost distribution of some tropical species and the southernmost distribution of certain temperate species overlap along this region, resulting in a unique species composition. The coastal habitats, such as seagrass beds or tidal mudflats, are of particular importance for this rich marine biodiversity (Coll et al. 2010; Unsworth and Cullen-Unsworth 2014). These habitats in the shallow, euphotic zone provide essential hiding places and food sources for many marine organisms and additionally serve as nurseries for numerous endangered and commercially exploited species (Jackson et al. 2015; Dewsbury et al. 2016). Understanding the interspecific relationships within these ecosystems is crucial to initiate necessary conservation measures in response to threats from climate change and urbanisation. Such understanding relies heavily on the taxonomical knowledge of locally occurring species. The relatively low sampling coverage along the Mauritanian and West African coast (Thyrring et al. 2024) has led to significant knowledge gaps concerning the invertebrate species diversity.

This study aims to provide an overview of the coastal shallow-water habitats of Mauritania, including seagrass beds, maerl beds, and tidal mudflats, and to catalogue the most abundant macrozoobenthic invertebrate species present in this environment additionally highlighting their most outstanding morphological characteristics.

## Materials and methods

### Study area

**Figure 1.**
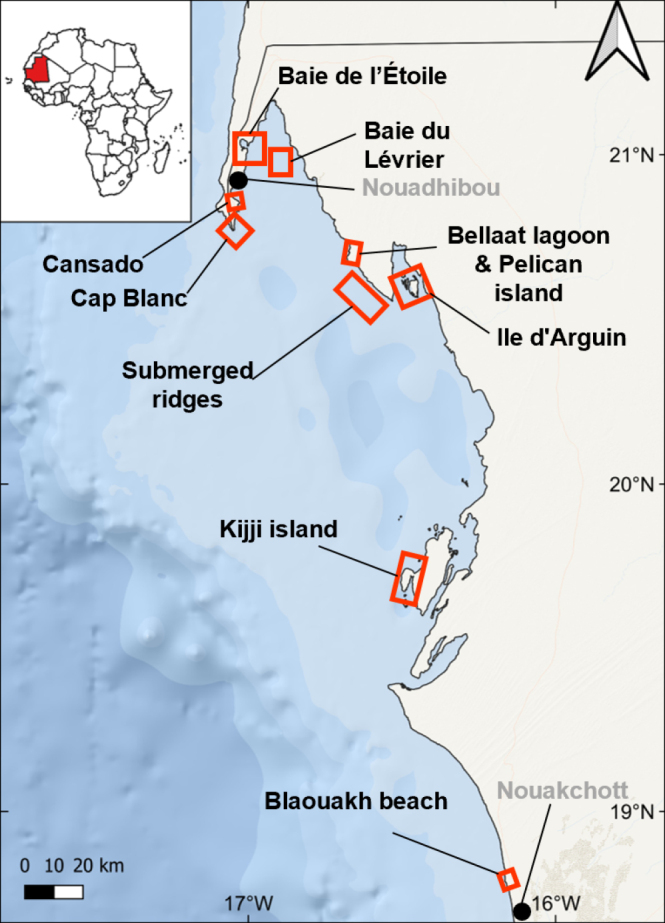
Geographical map of study areas along the North-Mauritanian coastline. Basemap from ESRI (2019, www.esri.com). Red boxes indicate the sampling locations.

**Figure 2.**
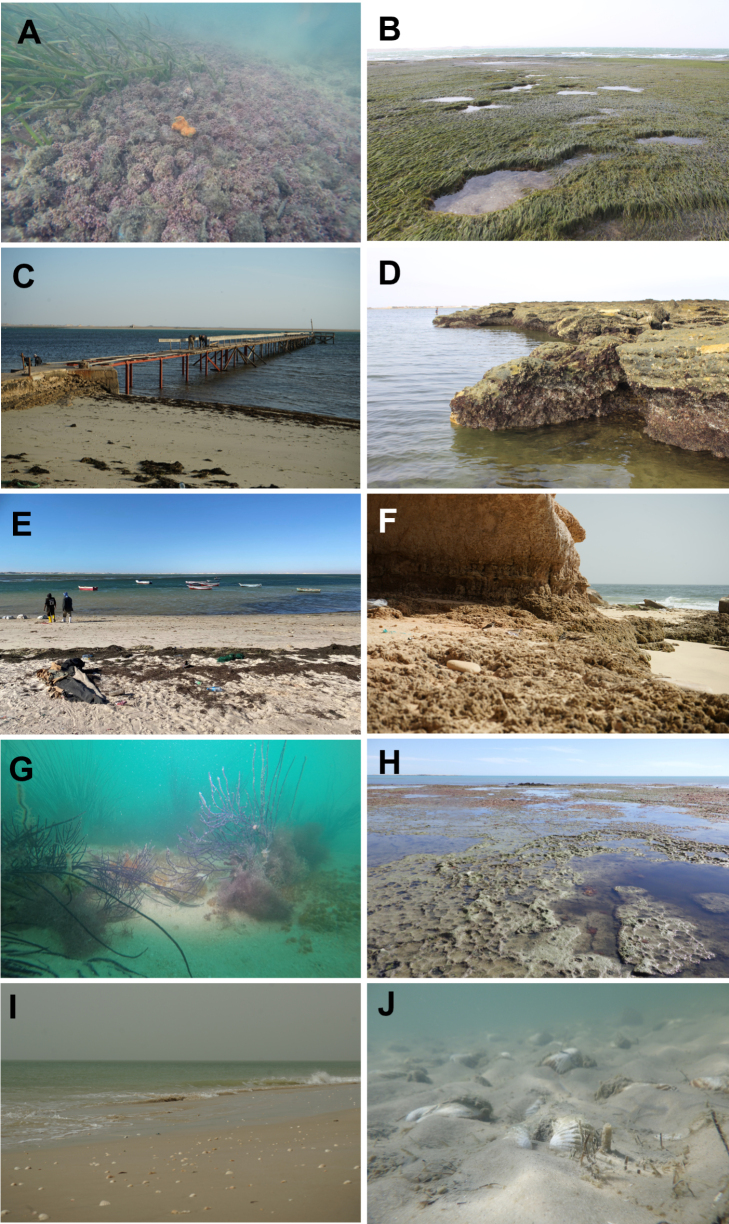
Overview of the studied habitats in Mauritania. **A**. Underwater image of the maerl ecosystem at the Baie de l’Étoile; **B**. *Nanozostera noltei* bed in the Baie de l’Étoile; **C**. The old pier at the “Centre de Pêche”, Baie de l’Étoile; **D**. Hard substrate of the rocky island in the Baie de l’Étoile; **E**. Artisanal fisheries landing place at the Baie de l’Étoile; **F**. Rocky shoreline at Cap Blanc; **G**. *Leptogorgia*-forest from the submerged ridge field; **H**. Sandstone tidepools at Pelican island in front of the Bellaat lagoon entrance; **I**. Sandy beach at the Blaouakh village; **J**. Sandy beach area at Agadir with great abundances of alive *Senilia senilis*. © Alexander Knorrn: A, G, © Kristina Hopf: C, F, I, © André Freiwald: D, E, H, © Friedhelm Krupp: B, © Moritz Sonnewald: J.

The study area comprises 17 different locations along the north Mauritanian coast (Figs 1, 2). Six areas (Bellaat lagoon, Bellaat lagoon entrance, Kijji island, Pelican island, the old stone pier as well as the sandy beaches of Agadir at the Ile d’Arguin (Fig. 2J), and a submerged ridge field) are situated within the UNESCO World Heritage Site Banc d’Arguin National Park (PNBA), where artisanal fishing is only permitted for the local Imraguen (an ethnic group of traditional fishermen from Mauritania) with non-motorised boats (Ly and David 2021). The Baie de l’Étoile, the area with the densest sample coverage for this study, was designated as a marine protected area during the 30 August 2024 by the Mauritanian Ministry of Environment and Sustainable Development. The seagrass beds of the Baie de l’Étoile consist of *Nanozostera noltei* (Hornemann, 1832) Tomlinson & Posluszny, 2001 (Fig. 2B) and *Cymodocea nodosa* (Ucria) Ascherson, 1870, while a hitherto undescribed maerl bed is located at the shallower areas of the Baie de l’Étoile (Fig. 2A). Additional hard substrates, including a rocky island (Fig. 2D) in the centre of the embayment and artificial structures such as an old fishing pier (Centre de Pêche, Fig. 2C) support local sessile faunal assemblages. Despite its newly acquired protection status, the Baie de l’Étoile is still subjected to significant anthropogenic stressors as a consequence of expanding urbanisation of Nouadhibou city. The ecosystem is heavily impacted by artisanal fisheries, waste water discharge, and plastic pollution. A similar situation applies to the following three areas Cap Blanc (Fig. 2F), Baie du Lévrier, Cansado beach, Blaouakh beach, which lack an official protection status but face similar anthropogenic threats. However, it must be noted that a small part of Cap Blanc functions as a marine protected area, as it is home to the southernmost population of the Mediterranean monk seal (*Monachus monachus*), which inhabits two caves at the southernmost tip, which are under protection (Badosa et al. 2006).

The Bellaat lagoon is a very young ecosystem formed by a dune breach during a heavy rain event in 2013 (Trégarot et al. 2020). This shallow water ecosystem resembles an undisturbed lagoon with extensive *N. noltei* assemblages and a sandy seafloor facing only a marginal human impact, except from the local Imraguen fishermen (Grebenart 2020; Ly and David 2021). Nearly no hard substrate is available within the Bellaat lagoon. The entrance area is a mainly sandy area with sparsely dispersed limestone aggregations.

The seagrass beds around Kijji island are located along a sandy area and rooted within an old oyster shell bed. Hard substrate habitats were investigated along the rocky shores of Cap Blanc, the sandstone tide pools of Pelican island (Fig. 2H) in front of the Bellaat lagoon entrance, the rocky shoreline at Cansado, an old stone pier at Agadir and the rocky island in the Baie de l’Étoile. The submerged ridge field off the Cap Sainte Anne coastal plain with its octocoral forest habitats was additionally investigated (Fig. 2G). These octocoral forests, formed by *Filigorgia* soft corals and large ascidian assemblages of *Stolonica cf. socialis*, were previously undescribed.

On various occasions, surveys were conducted at the artisanal fish landing sites in Nouadhibou (February 2023 and February 2024), the Baie de l’Étoile (August 2023, Fig. 2E), and Nouakchott (August 2022, June 2023, and January 2024). Additionally, the sandy beaches around the small village Blaouakh were sampled (Fig. 2I, Table 1).

**Table 1.** Sampling locations in shallow-water habitats and at artisanal landing places of northern Mauritania. Coordinates expressed in decimal degrees, only represent the centre of each location and do not indicate the exact sampling site of each species.

Habitat	Location	Latitude (°N), Longitude (°W)
Maerl bed	Baie de l’Étoile (M-BdE)	21.0209, -17.0058
	Pelican island (M-PI)	20.7111, -16.6880
Seagrass beds	Baie de l’Étoile (S-BdE)	21.0383, -17.0234
	Bellaat lagoon (S-BdL)	20.6919, -16.6703
	Kijji island (Kijji)	19.8227, -16.5940
Submerged ridge field	Submerged ridge field (SRF)	20.6531, -16.7267
Sandy seafloor	Blaouakh beach (BB)	18.5187, -16.0750
	Agadir sandy beach (ASB)	20.6106, -16.4468
	Baie du Lévrier (BdL)	20.9623, -16.8805
Hard substratum	Old Pier, Baie de l’Étoile (OP)	21.0207, -17.0046
	Rocky island, Baie de l’Étoile (RI)	21.0256, -17.0071
	Cap Blanc (CB)	20.7711, -17.0468
	Rocky shore Cansado (RSC)	20.8536, -17.0287
	Bellaat Lagoon entrance (BE)	20.6803, -16.6752
	Old Stone Pier at Agadir (OSP)	20.6125, -16.4472
Artisanal Fishermen	Nouakchott fish market (NKM)	18.1033, -16.0263
	Fish landing place in the Baie de l’Étoile (F-BdE)	21.0198, -17.0028

### Sampling

The marine biodiversity of the North-Mauritanian coastline has been documented through five expeditions conducted between 2020 and 2024 by the Institut Mauritanien de Recherche Océanographique et des Pêches (**IMROP**) and the Senckenberg Research Institute (**SRI**). Various sampling methods were applied, including hand collection, fish traps, beach seine sampling along the coastal habitats and artisanal fish market surveys from the land side, to cover a wide range of habitats. Additional sampling was conducted with a beam trawl and a “ganchorra” (a Portuguese dredge) from aboard the RV Amrigue along the submerged ridge field in front of the Cap Sainte Anne coastal plain. The collected living specimens were euthanised on crushed ice or with an overdose of clove oil (for cephalopods) and subsequently preserved for further identification and cataloguing for the scientific reference collections of both institutes. Specimens smaller than 10 cm were, in most cases, directly preserved in 70% ethanol, while larger specimens were initially preserved in formalin and gradually transferred to 70% ethanol after several weeks, according to Freyhof et al. (2020). Tissue samples were taken directly in the field and were preserved in 96% ethanol for later DNA barcoding. Several photographs of the studied specimens were taken in the field and laboratories. Subsequently, the collected specimens were morphologically identified (in most of the cases to species level) using specific identification literature. If a clear morphological identification was not possible, genetic sequence data was used to additionally validate the identification. Species new to Mauritania were marked as “First record” and their occurrences compared to currently available scientific literature and the Global Biodiversity Information Facility (GBIF, https://www.gbif.org/). Finally, all identified lots of species were separated into even parts and were catalogued for the scientific collections of IMROP and SRI. While the collections of SRI are already well established, the collections of IMROP were newly founded and currently are still hosted at SRI until the appropriate infrastructure at IMROP is built. The SRI collection metadata can be accessed via “https://search.senckenberg.de/aquila-public-search/search” or via “http://sesam.senckenberg.de/”, while the IMROP metadata portal will be moving to a more modern database before it will be publicly released. For this checklist, the ID numbers (following the codes “**SMF**” for SRI and “**IMROP**” for IMROP) indicate a representative lot of each species. These, along all the other lots are accessible via the above-mentioned meta-databases and can be made available upon request. While the data from the SRI collections are already mirrored to “https://www.gbif.org/” and “https://www.obis.org/”, this procedure still has to be established for IMROP. The generated DNA-barcodes are freely accessible via the Barcode Of Life Database (https://boldsystems.org/; Ratnasingham et al. 2024) as part of the Project “Mauritanian marine life barcode library (MAU)”. Another publication is highlighting the Mauritanian ichthyofauna collected along the same shallow-water habitats and complements the present study (Knorrn et al. 2025a).

## Results

### Species Identification

Morphological characteristics for all identified species were obtained from specific identification literature which is provided in the checklist underneath each species’ scientific name. The box “Identification” respectively is a citation of these sources for identification. A checklist of all documented species from each phylum and their associated habitats and locations can be seen in Table 2–9.

**Table 2.** Checklist of the marine Cnidaria from coastal habitats in Mauritania. Abbreviations provided in Table 1.

Species	Locations
Cnidaria (*n* = 2)	
Actiniidae	
*Actinia equina* (Linnaeus, 1758)	OP
*Anemonia sulcata* (Pennant, 1777)	M-BdE, RI

**Table 3.** Checklist of the marine Crustacea from coastal habitats and artisanal markets in Mauritania. Abbreviations provided in Table 1.

Species	Locations
Crustacea (*n* = 33)	
Sphaeromatidae	
*Sphaeroma serratum* (J. C. Fabricius, 1787)	OP
Talitridae	
*Orchestia mauritanica* Momtazi & Myers, 2025	BE
Chthamalidae	
*Chthamalus montagui* Southward, 1976	RI
Balanidae	
*Amphibalanus amphitrite* (Darwin, 1854)	RI
*Perforatus perforatus* (Bruguière, 1789)	RI
Pollicipedidae	
*Pollicipes pollicipes* (Gmelin, 1791)	CP
Palaemonidae	
*Palaemon elegans* Rathke, 1836	S-BdE
*Periclimenes sagittifer* (Norman, 1861)	KI
Palinuridae	
*Palinurus mauritanicus* Gruvel, 1911	NDM
*Panulirus regius* de Brito Capello, 1864	NDM
Penaeidae	
*Penaeus kerathurus* (Forskål, 1775)	NDM, S-BdE
*Penaeus notialis* Pérez Farfante, 1967	S-BdE, F-BDE
Porcellanidae	
*Pisidia bluteli* (Risso, 1816)	OP
*Pisidia longicornis* (Linnaeus, 1767)	RI, KI
*Porcellana platycheles* (Pennant, 1777)	RI, PI, SRF, OP
Diogenidae	
*Diogenes arguinensis* Almón, Cuesta & García-Raso, 2022	BdL
Paguridae	
*Pseudopagurus granulimanus* (Miers, 1881)	S-BdE, M-BdE, PI, SRF
Epialtidae	
*Pisa tetraodon* (Pennant, 1777)	OP, SRF
Leucosiidae	
*Atlantophila cristata* (Miers, 1881)	SRF
Inachidae	
*Macropodia linaresi* Forest & Zariquiey Álvarez, 1964	KI, SRF
Majidae	
*Maja brachydactyla* Balss, 1922	OP, SRF, M-BdE
Oziidae	
*Eupilumnus stridulans* (Monod, 1956)	S-BdE, RI
Pilumnidae	
*Serenepilumnus pisifer* (MacLeay, 1838)	SRF, KI
Portunidae	
*Callinectes amnicola* (de Rochebrune, 1883)	S-BdL
*Callinectes marginatus* (A. Milne-Edwards, 1861)	S-BdE
*Thalamita poissonii* (Audouin, 1826)	S-BdE, OP
Carcinidae	
*Carcinus maenas* (Linnaeus, 1758)	S-BdE
Panopeidae	
*Panopeus africanus* A. Milne-Edwards, 1867	S-BdE, RI, PI
Xanthidae	
*Xantho poressa* (Olivi, 1792)	OP
Grapsidae	
*Pachygrapsus transversus* (Gibbes, 1850)	OP
Varunidae	
*Dudekemus atlanticus* (Monod, 1933)	BdL
Ocypodidae	
*Afruca tangeri* (Eydoux, 1835)	S-BdE, S-BdL, IdA
Pinnotheridae	
*Pinnotheres pisum* (Linnaeus, 1767)	OP

**Table 4.** Checklist of the marine Polyplacophora from shallow-water habitats in Mauritania. Abbreviations explained in Table 1.

Species	Location
Polyplacophora (*n* = 2)	
Acanthochitonidae	
*Acanthochitona fascicularis* (Linnaeus, 1767)	KI
Ischnochitonidae	
*Ischnochiton cessaci* (Rochebrune, 1881)	KI

**Table 5.** Checklist of the marine Gastropoda from shallow-water habitats in Mauritania. Abbreviations explained in Table 1.

Species	Location
Gastropoda (*n* = 28)	
Patellidae	
*Patella rustica* Linnaeus, 1758	CB
*Patella depressa* Pennant, 1777	RSC
Fissurellidae	
*Diodora graeca* (Linnaeus, 1758)	SRF
Trochidae	
*Phorcus lineatus* (da Costa, 1778)	RI, OP, S, PI, CB, IdA
*Steromphala umbilicalis* (da Costa, 1778)	M-BdE, OP, IdA
Turritellidae	
*Mesalia mesal* (Deshayes, 1843)	SRF, S-BdE
*Mesalia opalina* (A. Adams & Reeve, 1849)	SRF
Calyptraeidae	
*Crepidula porcellana* Lamarck, 1801	M-BdE, S-BdE, OP, SRF, PI, IdA
Cypraeidae	
*Zonaria zonaria* (Gmelin, 1791)	SRF
Naticidae	
*Natica fulminea* (Gmelin, 1791)	KI, SRF, S-BdE
Muricidae	
*Bolinus cornutus* (Linnaeus, 1758)	S-BdE
*Hexaplex rosarium* (Röding, 1798)	M-BdE, OP
*Stramonita haemastoma* (Linnaeus, 1767)	RSC
Melongenidae	
*Pugilina morio* (Linnaeus, 1758)	S-BdE, M-BdE, PI, OP
Nassariidae	
*Tritia pfeifferi* (R. A. Philippi, 1844)	PI, S-BdE
Columbellidae	
*Columbella adansoni* Menke, 1853	OP
*Mitrella broderipii* (G. B. Sowerby I, 1844)	M-BdE
Pisaniidae	
*Aplus assimilis* (Reeve, 1846)	OP, RI
Volutidae	
*Cymbium marmoratum* Link, 1807	F-BdE, RI
Marginellidae	
*Marginella cleryi* Petit de la Saussaye, 1836	SRF, S-BdE
*Marginella glabella* (Linnaeus, 1758)	S-BdE
*Volvarina ampelusica* Monterosato, 1906	SRF, S-BdE
Cystiscidae	
*Persicula cingulata* (Dillwyn, 1817)	KI, S-BdE
Conidae	
*Conus byssinus* (Röding, 1798)	M-BdE, S-BdE
Bullidae	
*Bulla striata* Bruguière, 1792	S-BdE, PI
Aplysiidae	
*Bursatella leachii* Blainville, 1817	SRF, S-BdE
Onchidiidae	
*Onchidella celtica* (Audouin & Milne-Edwards, 1832)	RI
Siphonariidae	
*Siphonaria pectinata* (Linnaeus, 1758)	RI, OP, IdA

**Table 6.** Checklist of the marine Bivalvia from shallow-water habitats and artisanal fish markets in Mauritania. Abbreviations are explained in Table 1.

Species	Location
Bivalvia (*n* = 28)	
Nuculanidae	
*Lembulus bicuspidatus* (A. Gould, 1845)	BdL
Arcidae	
*Acar olivercoseli M*. Huber, 2010	KI
*Senilia senilis* (Linnaeus, 1758)	KI, IdA
Mytilidae	
*Leiosolenus aristatus* (Dillwyn, 1817)	RI
*Perna perna* (Linnaeus, 1758)	RI, CB
Pinnidae	
*Atrina chautardi* (Nicklès, 1953)	S-BdE
Pectinidae	
*Aequipecten flabellum* (Gmelin, 1791)	BB
Ostreidae	
*Magallana gigas* (Thunberg, 1793)	S-BdE
*Ostrea stentina* Payraudeau, 1826	RI
Crassatellidae	
*Crassatina alba* Cosel, 1995	SRF
*Crassatina guineensis* Cosel & Gofas, 2018	SRF
Lucinidae	
*Lucinoma borealis* (Linnaeus, 1767)	BdL
Cardiidae	
*Cerastoderma edule* (Linnaeus, 1758)	S-BdE, SRF
*Papillicardium papillosum* (Poli, 1791)	KI, SRF
Tellinidae	
*Gastrana matadoa* (Gmelin, 1791)	S-BdE
*Huberimactra inconstans* (Cosel, 1995)	SRF
Psammobiidae	
*Gari depressa* (Pennant, 1777)	S-BdE, SRF
*Gari fervensis* (Gmelin, 1791)	SRF
*Gari jousseaumeana* Bertin, 1880	BdL
Solenidae	
*Solen capensis* P. Fischer, 1881	S-BdE
Veneridae	
*Callista floridella* (Gray, 1838)	KI, SRF
*Petricola lithophaga* (Retzius, 1788)	RI, SRF
*Pitar tumens* (Gmelin, 1791)	SRF
*Polititapes durus* (Gmelin, 1791)	SRF, KI
*Ruditapes decussatus* (Linnaeus, 1758)	S-BdE
*Venerupis corrugata* (Gmelin, 1791)	SRF, IdA
*Venus casina* Linnaeus, 1758	SRF, BdL
*Venus verrucosa* Linnaeus, 1758	S-BdE, M-BdE, SRF

**Table 7.** Checklist of the Cephalopoda from shallow-water habitats and artisanal fish markets in Mauritania. Abbreviations provided in Table 1.

Species	Location
Cephalopoda (*n* = 2)	
Sepiidae	
*Sepia officinalis* Linnaeus, 1758	S-BdE
Ommastrephidae	
*Todarodes sagittatus* (Lamarck, 1798)	NKM

**Table 8.** Checklist of the Brachiopoda species from shallow-water habitats in Mauritania. Abbreviations provided in Table 1.

Species	Location
Brachiopoda (*n* = 1)	
Lingulidae	
*Lingula parva* Smith, 1872	BdL, KI

**Table 9.** Checklist of the marine Echinodermata from shallow-water habitats and artisanal fish markets in Mauritania. Abbreviations provided in Table 1.

Species	Location
Echinodermata (*n* = 7)	
Asterinidae	
*Asterina gibbosa* (Pennant, 1777)	M-BdE
*Asterina stellifera* (Möbius, 1859)	PI
Amphiuridae	
*Amphipholis squamata* (Delle Chiaje, 1828)	M-BdE
Ophiactidae	
*Ophiactis lymani* Ljungman, 1872	RI, M-BdE
Ophiotrichidae	
*Ophiothrix cotteaui* (de Loriol, 1900)	SRF
Arbaciidae	
*Arbacia lixula* (Linnaeus, 1758)	BB
Holothuriidae	
Holothuria (Roweothuria) arguinensis Koehler & Vaney, 1906	BB

### Abbreviations

**SMF** [accession number] = reference collections of the Senckenberg Research Institute;

**IMROP** [accession number] = reference collections of the Institut Mauritanien de Recherche Océanographique et des Pêches;

**MAU** [accession number] = reference to according genetic sequence data stored in BOLD-Systems project “MAU Mauritanian marine life barcode library”.

### Checklist

#### Cnidaria

***Actinia equina* (Linnaeus, 1758)**

**References**. Riedl (1983); Hayward and Ryland (2017).

**First record**. MAURITANIA - Dakhlet Nouadhibou • Old Pier; 21.0199, -17.0055; 24.VII.2022; A. Knorrn, F. Krupp, S. M. Moctar, and A. Freiwald leg.; collected from stones in close proximity to an old pier; BdE-02; IMROP 3, MAU-256.

**Identification**. Solitary specimen with distinct adhering disc. Smooth column with a relatively equal height and diameter. Up to 196 tentacles without distinct pattern and terminal knobs, arranged in six cycles around the mouth opening. Verrucae absent. Body uniformly reddish, greenish, or brownish coloured.

***Anemonia sulcata* (Pennant, 1777)**

**References**. Riedl (1983); Hayward and Ryland (2017).

**Records**. MAURITANIA - Dakhlet Nouadhibou • Maerl bed in the Baie de l’Étoile; 21.0254, -17.0073; 26.VII.2022; A. Knorrn, F. Krupp, S. M. Moctar, and A. Freiwald leg.; hand collection; BdE-20-Maerl; SMF 13117 • Rocky island; 21.0252, -17.0069; 18.II.2023; A. Knorrn, M. Sonnewald, S. M. Moctar, and A. Freiwald leg.; hand collection; BdE-102; IMROP 2.

**Identification**. Body typically wider than high. Approximately 200 long and sinuous tentacles, which are rarely retracted. Inconspicuous acrorhagi on the parapet. Column usually brownish to bright brownish in colouration with brownish, greyish, or even bright green tentacles with purple tips.

#### Crustacea

***Sphaeroma serratum* (J. C. Fabricius, 1787)**

**References**. Naylor (1972).

**First record**. MAURITANIA - Dakhlet Nouadhibou • Old Pier; 21.0197, -17.0054; 02.XII.2021; M. Sonnewald, S. M. Moctar, and A. Freiwald leg.; collected underneath stones at beginning of an old pier; CDP21-02-2; IMROP 1, SMF 58179.

**Identification**. Slightly acute pleotelson and antennulae with 9–15 aesthetascs. Ischium of pereopod I covered with ≤ 60 plumose setae, which are arranged in two rows. Uropods with 4–7 well defined serrations on the outer edge of the exopod. Various in colouration, ranging between a reddish to brownish to dark greyish colouration.

***Orchestia mauritanica* Momtazi & Myers, 2025**

**References**. Momtazi et al. (2025).

**Record**. MAURITANIA - Dakhlet Nouadhibou • Bellaat Lagoon entrance; 20.6803, -16.6752; 03.III.2023; M. Sonnewald, A. Knorrn, S. M. M. Moctar, and A. Freiwald leg.; collected underneath litoral rocks; BdL-61; IMROP 60, SMF 62512.

**Identification**. Amphipod with irregular carpus in the pereopod VII and extremely acute palm in the second male gnathopod. The straight distal posterior propodus margin, a distinct proximal shelf on the second male gnathopod and the lack of concavity on the posterior margin of the carpus in the pereopod VII characterise the species.

***Chthamalus montagui* Southward, 1976**

Fig. 3A

**Figure 3.**
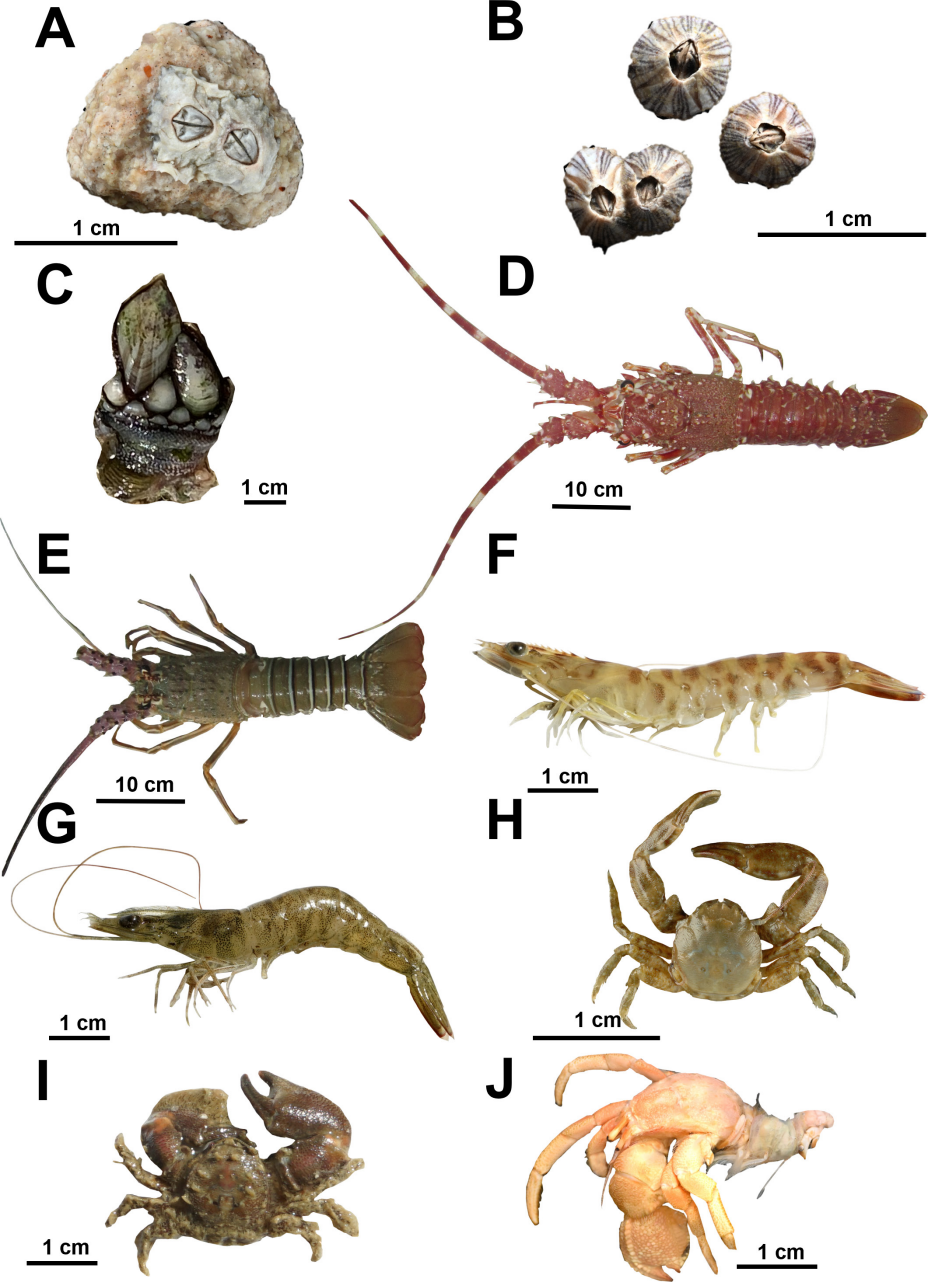
Marine crustaceans from coastal habitats in northern Mauritania. **A**. *Chthamalus montagui*; **B**. *Amphibalanus amphitrite*; **C**. *Pollicipes pollicipes*; **D**. *Palinurus mauritanicus*; **E**. *Panulirus regius*; **F**. *Penaeus kerathurus*; **G**. *Penaeus notialis*; **H**. *Pisidia longicornis*; **I**. *Porcellana platycheles*; **J**. *Pseudopagurus granulimanus*. © Alexander Knorrn: A, G, © Nicol Mahnken: A, B, © Kristina Hopf: F, G, I, © Moritz Sonnewald: C, J, © Mamadou Dia: D, E, © Sven Tränckner: H.

**References**. Southward (2008); Hayward and Ryland (2017).

**First record**. MAURITANIA - Dakhlet Nouadhibou • Rocky island; 21.0256, -17.0071; 26.VII.2022; A. Knorrn, F. Krupp, S. M. Moctar, and A. Freiwald leg.; from littoral rocks; BdE-20; IMROP 35; SMF 61106, MAU-245.

**Identification**. Kite-shaped operculum and a junction between tergum and scutum close to the carinal edge. Scutum longer than broad. Brownish or greyish colouration. Surface often corroded with obliterated sutures.

**Remarks**. This species is currently only known from the Mediterranean coasts of Italy, France, and Spain, from the North-East Atlantic coast of Portugal, Spain, France, and all coasts of the UK and Ireland.

***Amphibalanus amphitrite* (Darwin, 1854)**

Fig. 3B

**References**. Darwin (1854).

**Record**. MAURITANIA - Dakhlet Nouadhibou • Rocky island; 21.0254, -17.0067; 17.II.2023; A. Knorrn, M. Sonnewald, S. M. Moctar, and A. Freiwald leg.; from intertidal rocks; BdE-64; IMROP 44, SMF 61116, MAU-460.

**Identification**. Scutum internally with a prominent broad adductor ridge. Shell longitudinally striped with purple or pink colouration, sometimes with confluent stripes or wholly white in colour.

***Perforatus perforatus* (Bruguière, 1789)**

**References**. Southward (2008); Hayward and Ryland (2017).

**Record**. MAURITANIA - Dakhlet Nouadhibou • Rocky island; 21.0256, -17.0071; 26.VII.2022; A. Knorrn, F. Krupp, S. M. Moctar, and A. Freiwald leg.; from littoral rocks; BdE-20; SMF 61094, MAU-244.

**Identification**. Body in regular conical shape and with a relatively small opercular opening. Scutum shows moderate growth ridges but without longitudinal striations. A dark purple ground colour with additional patches of blue and white and sometimes even pink.

***Pollicipes pollicipes* (Gmelin, 1791)**

Fig. 3C

**References**. Hayward and Ryland (2017).

**First record**. MAURITANIA - Dakhlet Nouadhibou • Cap Blanc; 20.7710, -17.0467; 23.II.2023; A. Knorrn, S. M. Moctar, M. Sonnewald, and A. Freiwald leg.; hand collection from rocky shoreline; BdL-22; IMROP 51, SMF 61123.

**Identification**. Capitulum laterally compressed with five prominent plates and a variable number of smaller spine-like plates, all with terminal umbones. Surface of peduncle is armoured with uniformly small plates. Peduncle usually dark brown to reddish in colour with ivory to brownish white plates.

***Palaemon elegans* Rathke, 1836**

**References**. Hayward and Ryland (2017).

**Record**. MAURITANIA - Dakhlet Nouadhibou • Seagrass bed in the Baie de l’Étoile; 21.0273, -17.0042; 26.VII.2022; A. Knorrn, S. M. Moctar, and A. Freiwald leg.; Beach seine; BdE-16; IMROP 28, SMF 61097, MAU-234.

**Identification**. Rostrum straight with 7–9 dorsal teeth, three ventral teeth, three of the dorsal teeth are located behind the posterior edge of the orbit. Carpus region of pereopod II of not subdivided. First pereopod with a well-developed chela. Body uniformly greyish with a dark yellow to brownish band around the thorax and pleon.

***Periclimenes sagittifer* (Norman, 1861)**

**References**. Grippa et al. (1996).

**First record**. MAURITANIA - Dakhlet Nouadhibou • Kijji island; 19.8227, -16.5940; 20.VI.2023; A. Knorrn and A. Freiwald leg.; ganchorra haul; K001; IMROP 57, SMF 61129.

**Identification**. Body and walking legs quite robust. Rostrum very high: 2.8-3.4× longer than high. Second pereopods distinctly unequal with a short carpus, < 2× longer than broad. Fingers of the large second pereiopod shorter than palm.

**Remarks**. Currently, only known from the North-East Atlantic coasts of Portugal, Spain, France, and from the English Channel, the Azores, and from Lanzarote as the southernmost tip of its distribution.

***Palinurus mauritanicus* Gruvel, 1911**

Fig. 3D

**References**. Carpenter and De Angelis (2016a).

**Record**. MAURITANIA - Dakhlet Nouadhibou • Nouadhibou Market; 20.9121, - 17.0425; 21.II.2022; M. Dia and A. Niang leg.; fish market survey (tissue sample); MAU-71.

**Identification**. Carpus of the pereopod I without anterodorsal spine. Anterior border of carapace bearing two strong, rather wide, externally convex, frontal horns. Tips separated by slightly concave margin armed with several denticles. Anterior margin of carapace not deeply concave between the frontal horns. Reddish or pinkish body colouration with whitish marbling and spots over the entire dorsal surface. Legs with several irregular pinkish or white spots.

***Panulirus regius* de Brito Capello, 1864**

Fig. 3E

**References**. Carpenter and De Angelis (2016a).

**Record**. MAURITANIA - Dakhlet Nouadhibou • Nouadhibou Market; 20.9121, -17.0425; 21.II.2022; M. Dia and A. Niang leg.; fish market survey (tissue sample); MAU-68.

**Identification**. Abdominal segments with shallow transverse grooves filled with short hairs and broadly interrupted in midline. Body generally coloured in various shades of green. Each tail segment with a white transverse band, separated from the posterior margin by a greenish to brownish band. Several small whitish spots on the pleura bases of each abdominal segment.

***Penaeus kerathurus* (Forskål, 1775)**

Fig. 3F

**References**. Carpenter and De Angelis (2016a).

**Records**. MAURITANIA - Dakhlet Nouadhibou • Nouadhibou Market; 20.9121, -17.0425; 01.II.2024; M. Dia and A. Niang leg.; fish market survey (tissue sample); MAU-392 • Seagrass bed in the Baie de l’Étoile; 21.0168, -17.0184; 01.II.2024; A. Knorrn, M. Sonnewald, S. M. Moctar, and A. Freiwald leg.; Beach seine; BdE-227; SMF 61117.

**Identification**. Rostrum slightly curved upwards at the tip. Usually 11 (8–13) teeth on the dorsal side and one single and strongly pronounced tooth on the ventral margin. Males with pink bars on the abdomen. Females are greyish or yellowish coloured with copper-green or brownish bars. Tail fan sometimes blueish towards the tip and edged with red.

**Remarks**. Easy to be distinguished from *P. notialis* by the presence of only a single tooth on the ventral margin of the rostrum.

***Penaeus notialis* Pérez Farfante, 1967**

Fig. 3G

**References**. Carpenter and De Angelis (2016a).

**Records**. MAURITANIA - Dakhlet Nouadhibou • Seagrass bed in the Baie de l’Étoile; 21.0383, -17.0234; 27.VII.2022; A. Knorrn, M. Sonnewald, S. M. Moctar, and A. Freiwald leg.; Beach seine; BdE-27; IMROP 45, SMF 61118 • Fishermen from the Baie de l’Étoile; 21.0198, -17.0028; 09.III.2023; A. Knorrn, S. M. Moctar, and A. Freiwald leg.; in front of the BdE opening; gill net of local fishermen (tissue sample); MAU-464.

**Identification**. Rostrum usually with nine (8–11) teeth on the dorsal side and two strong teeth on the ventral side. Three scars present laterally. Dorsolateral grooves on the sixth abdominal segment are well-defined and broad. Body uniformly blond to orange coloured. Mostly with a dark blotch at the junction of the third and fourth abdominal segments.

**Remarks**. Easy to be distinguished from *P. kerathurus* by the presence of two strong teeth on the ventral margin of the rostrum.

***Pisidia bluteli* (Risso, 1816)**

**References**. Ferreira and Tavares (2020).

**Record**. MAURITANIA - Dakhlet Nouadhibou • Old Pier; 21.0206, -17.0046; 02.XII.2021; M. Sonnewald, S. M. Moctar, and A. Freiwald leg.; collected underneath an old pier; CDP21-01-3; SMF 58347.

**Identification**. Almost circular carapace with several distinct spines along the dorsal part of the carapace. A trilobed front with a median and with longitudinal furrows that are serrated terminally. A very long and slender antenna and unequal chelipeds. Body colouration dark brownish to olive.

**Remarks**. Easy to be distinguished from *P. longicornis* by its thorned meri.

***Pisidia longicornis* (Linnaeus, 1767)**

Fig. 3H

**References**. Hayward and Ryland (2017); Ferreira and Tavares (2020).

**Records**. MAURITANIA - Dakhlet Nouadhibou • Rocky island; 21.0248, -17.0071; 17.II.2023; A. Knorrn and A. Freiwald leg.; fish trap; BdE-63; SMF 61121 • Kijji island; 19.8249, -16.5954; 20.VI.2023, A. Knorrn leg.; hand collection while diving; K003; SMF 62514, MAU-467.

**Identification**. Almost circular carapace, slightly convex with no setae. A trilobed front with a median and with longitudinal furrows that are serrated terminally. Antenna very long and slender with unequal chelipeds. Body colouration dark brownish to olive.

**Remarks**. Easy to be distinguished from *P. bluteli* by its not thorny meri.

***Porcellana platycheles* (Pennant, 1777)**

Fig. 3I

**References**. Hayward and Ryland (2017).

**Records**. MAURITANIA - Dakhlet Nouadhibou • Rocky island; 21.0259, -17.0069; 17.II.2023; A. Knorrn and A. Freiwald leg.; hand collection; BdE-65; SMF 61124 • Pelican island; 20.7111, -16.6880; 03.III.2023; A. Knorrn, M. Sonnewald, S. M. Moctar, and A. Freiwald leg.; hand collection from a maerl and rock habitat; BdL-74; IMROP 53, SMF 61125 • Submerged ridges; 20.6632, -16.7047; 04.II.2024; A. Knorrn, S. M. Moctar, M. Sonnewald, and A. Freiwald leg.; ganchorra haul; BdL-124; SMF 62527 • Old Pier; 21.0197, -17.0054; 30.XI.2021; M. Sonnewald, S. M. Moctar, and A. Freiwald leg.; collected underneath an old pier; CDP21-00; IMROP 5, SMF 58341, MAU-220.

**Identification**. Nearly circular carapace, slightly longer than broad with a setose posterior margin. Front of carapace with advanced median tooth flanked by smaller submarginals. Chelipeds strongly pronounced, unequal and compressed. The pereopods and distal edges of the abdominal segments are setose. Greyish to brownish coloured dorsally and yellowish white ventrally.

***Diogenes arguinensis* Almón, Cuesta & García-Raso, 2022**

**References**. Almón et al. (2022).

**Record**. MAURITANIA - Dakhlet Nouadhibou • Baie du Lévrier; 21.0135, -16.9611; 04.XII.2021; S. M. Moctar, and A. Freiwald leg.; grab sample; BdL 3-2; SMF 62520, MAU-225.

**Identification**. Carapace subquadrate, longer than broad, not vaulted. Rostral lobe narrowly rounded, exceeded by lateral triangular projections, acutely pointed, with a single spine in the apex. Chelae unequal, with the larger one on the left side. Palm on left cheliped higher than long.

***Pseudopagurus granulimanus* (Miers, 1881)**

Fig. 3J

**References**. Forest (1952).

**Records**. MAURITANIA - Dakhlet Nouadhibou • Seagrass bed in the Baie de l’Étoile; 21.0191, -17.0063; 24.VII.2022; A. Knorrn, M. Sonnewald, S. M. Moctar, and A. Freiwald leg.; hand collection; BdE-05; IMROP 32, SMF 61103, MAU-235 • Maerl bed in the Baie de l’Étoile; 21.0254, -17.0067; 17.II.2023; A. Knorrn, M. Sonnewald, S. M. Moctar, and A. Freiwald leg.; hand collection; BdE-64; IMROP 46, MAU-461 • Pelican island; 20.7085, -16.6839, 03.III.2023, A. Knorrn, M. Sonnewald, S. M. Moctar, and A. Freiwald leg.; hand collection from rock habitat; BdL-73; IMROP 47 • Submerged ridges; 20.6547, -16.7260; 02.III.2023; A. Knorrn, S. M. Moctar, M. Sonnewald, and A. Freiwald leg.; ganchorra haul; BdL-53; SMF 61119.

**Identification**. Left cheliped much larger than the right. From a certain size onward, the external surface of the forceps has the appearance of a mosaic of smooth, polygonal, or rounded plates, which does not allow any specific confusion.

***Pisa tetraodon* (Pennant, 1777)**

Fig. 4A

**Figure 4.**
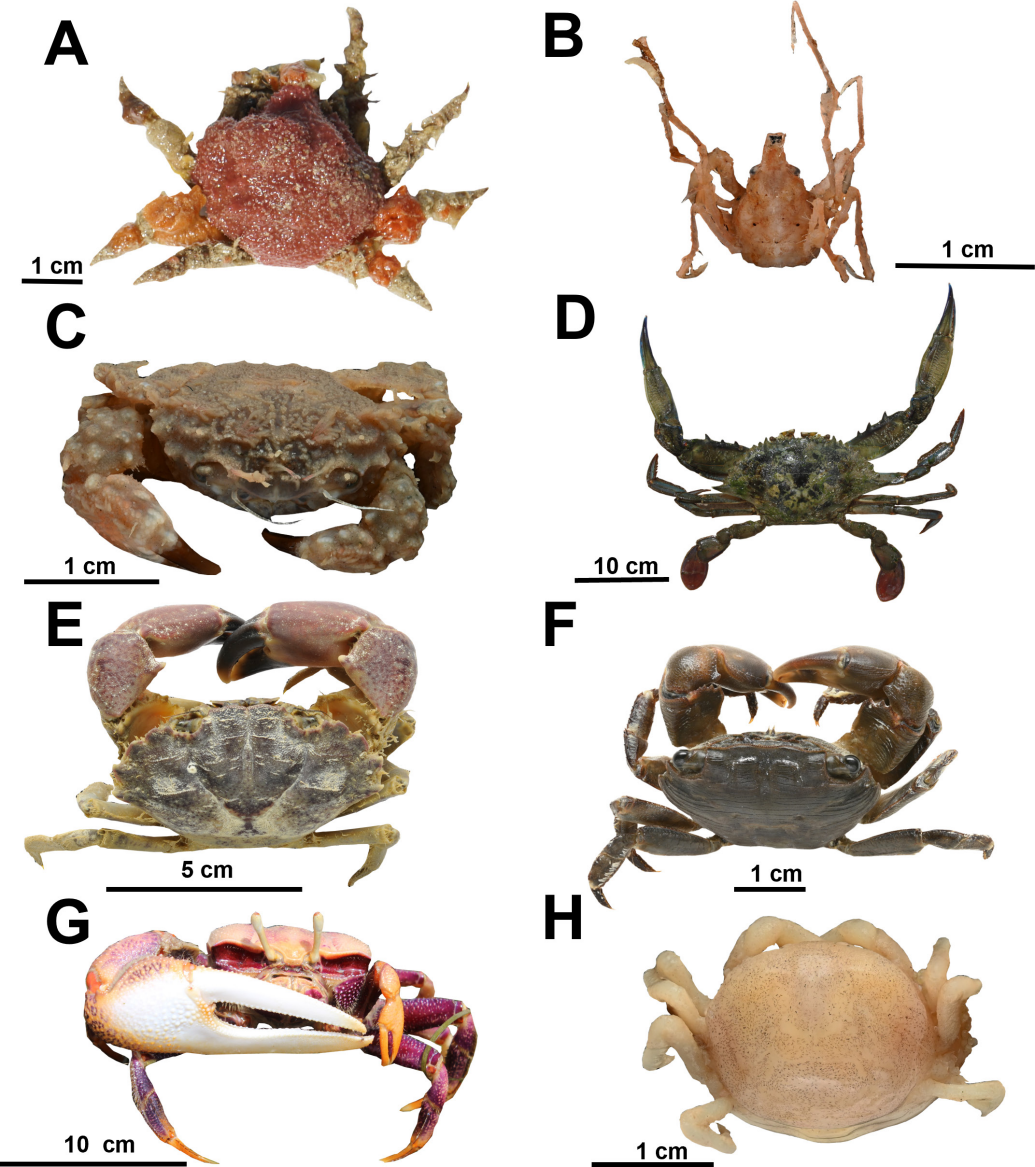
Marine crustaceans from coastal habitats in northern Mauritania. **A**. *Pisa tetraodon*; **B**. *Macropodia linaresi*; **C**. *Serenepilumnus pisifer*; **D**. *Callinectes marginatus*; **E**. *Panopeus africanus*; **F**. *Pachygrapsus transversus*; **G**. *Afruca tangeri*; **H**. *Pinnotheres pisum*. © Nicol Mahnken: B, C, E, F, H, © Kristina Hopf: A, © Friedhelm Krupp: D, G.

**References**. Monod (1956); Hayward and Ryland (2017).

**Records**. MAURITANIA - Dakhlet Nouadhibou • Old Pier; 21.0207, -17.0046; 19.II.2023; A. Knorrn, M. Sonnewald, S. M. Moctar, and A. Freiwald leg.; fish trap; BdE-103-2; IMROP 30, SMF 61100, MAU-396. • Submerged ridges; 20.6562, -16.7199; 04.II.2024; A. Knorrn, S. M. Moctar, M. Sonnewald, and A. Freiwald leg.; ganchorra haul; BdL-122; IMROP 55, SMF 61127.

**Identification**. Three large spines on each lateral side between post-orbitalis and post-lateralis. Posterior-median spine reduced and pre-orbital spine larger than the other spines. Body colouration brownish red.

***Atlantophila cristata* (Miers, 1881)**

**References**. Muñoz et al. (2024).

**Record**. MAURITANIA - Dakhlet Nouadhibou • Submerged ridges; 20.6531, -16.7267; 02.III.2023; A. Knorrn, S. M. Moctar, M. Sonnewald, and A. Freiwald leg.; ganchorra haul; BdL-51; SMF 62519, MAU-463.

**Identification**. Elongated and subcylindrical carapace with pointed apex. Oval and small aperture, located near the anterior end. Surface smooth with fine longitudinal lines, and there is no distinct umbilicus. Carapace reddish brown with darker striations.

***Macropodia linaresi* Forest & Zariquiey Álvarez, 1964**

Fig. 4B

**References**. van Noort and Adema (1985).

**Record**. MAURITANIA - Dakhlet Nouadhibou • Kijji island; 19.8227, -16.5940; 20.VI.2023; A. Knorrn and A. Freiwald leg.; ganchorra haul; K001; SMF 62513, MAU-466 • Submerged ridges; 20.6648, -16.7050; 04.II.2024; A. Knorrn, S. M. Moctar, M. Sonnewald, and A. Freiwald leg.; ganchorra haul; BdL-124; IMROP 61.

**Identification**. Slender and elongated carapace, with long, spindly legs. Carapace covered with fine granules and lacks distinct ridges. Chelae slender and slightly unequal. Rostrum very short and curved upwards. Rostrum does not extend beyond the proximal quarter of fifth segment of the antennal peduncle. Dactylus of the pereopod V is sickle-shaped, with long spines over the whole inner margin. Body is reddish brown with numerous fine white spots

***Maja brachydactyla* Balss, 1922**

**References**. Carpenter and De Angelis (2016a).

**Records**. MAURITANIA - Dakhlet Nouadhibou • Old Pier; 21.0206, -17.0046; 02.XII.2021; M. Sonnewald, S. M. Moctar, and A. Freiwald leg.; fish trap; CDP21-1-3; SMF 58350, MAU-218 • Submerged ridges; 20.6531, -16.7267; 02.III.2023; A. Knorrn, S. M. Moctar, M. Sonnewald, and A. Freiwald leg.; ganchorra haul; BdL-51; SMF 61120 • Maerl bed in the Baie de l’Étoile; 21.0202, -17.0046; 20.II.2023; A. Knorrn, M. Sonnewald, S. M. Moctar, and A. Freiwald leg.; hand collection; BdE-117; IMROP 24.

**Identification**. Carapace strongly vaulted with its greatest width behind the middle of the body. Anterior part of carapace ends with two strong and divergent teeth. Walking legs without any spines. Body rather uniformly reddish brown or yellowish brown.

***Eupilumnus stridulans* (Monod, 1956)**

**References**. Carpenter and De Angelis (2016a).

**First records**. MAURITANIA - Dakhlet Nouadhibou • Seagrass bed in the Baie de l’Étoile; 21.0191, -17.0063; 24.VII.2022; A. Knorrn, S. M. Moctar, and A. Freiwald leg.; hand collection during low tide; BdE-5; IMROP 56 • Rocky island; 21.0254, -17.0067; 17.II.2023; A. Knorrn and A. Freiwald leg.; hand collection; BdE-64; IMROP 63 • Submerged ridges; 20.6531, -16.7267; 02.III.2023; A. Knorrn, S. M. Moctar, M. Sonnewald, and A. Freiwald leg.; ganchorra haul; BdL-51; SMF 62519.

**Identification**. Body with sparse cover of setae. Median notch very shallow with no spines. Black part of male chela does not extend on the palm. Male abdomen with seven movable segments. Second male gonopod longer than the first one. Distal part of the second gonopod developed into a filiform flagellum.

***Serenepilumnus pisifer* (MacLeay, 1838)**

Fig. 4C

**References**. Smith (1838).

**Record**. MAURITANIA - Dakhlet Nouadhibou • Submerged ridges; 20,6878, -16,7131; 04.III.2023; A. Knorrn, S. M. Moctar, M. Sonnewald, and A. Freiwald leg.; ganchorra haul; BdL-86; SMF 62515, IMROP 62, MAU-571 • Kijji island; 19.8227, -16.5940; 20.VI.2023; A. Knorrn and A. Freiwald leg.; ganchorra haul; K001; SMF 61128.

**Identification**. Carapace convex in the middle part. Anterolateral margin with four blunt tubercles. Sub-acuminate front with crenated apex. Chela large and slightly unequal in size. Body bright brownish.

***Callinectes amnicola* (de Rochebrune, 1883)**

**References**. Carpenter and De Angelis (2016a).

**Record**. MAURITANIA - Dakhlet Nouadhibou • Seagrass bed in the Bellaat lagoon; 20.6803, -16.675285; 03.III.2023; A. Knorrn, S. M. Moctar, M. Sonnewald and A. Freiwald leg.; hand collection during low tide; BdL-62; IMROP 19; SMF 61088, MAU-388.

**Identification**. Carapace broad, ending laterally in a strong spine. Eight distinct teeth at lateral margin. Length of the lateral spine twice or more than that of the preceding teeth. Epibranchial ridge almost straight. Several granules along the carapace with ridges anterior to epibranchial larger and placed wider apart than those posterior to ridges. Olive brown body colouration with bluish colour at the anterior parts of the pereopod I.

***Callinectes marginatus* (A. Milne-Edwards, 1861)**

Fig. 4D

**References**. Carpenter and De Angelis (2016a).

**Record**. MAURITANIA - Dakhlet Nouadhibou • Seagrass bed in the Baie de l’Étoile; 21.0168, -17.0184; 25.VII.2022; A. Knorrn, F. Krupp, S. M. Moctar, and A. Freiwald leg.; Beach seine; BdE-07; SMF 61075.

**Identification**. Carapace broad and coarsely granulated, ending laterally in a strong spine. Eight distinct teeth at the lateral margin. Length of the lateral spine twice or more than that of the preceding teeth. Epibranchial ridges with a distinct inflection in the middle. First abdominal somite ends laterally in a triangular point, which is neither sharply drawn nor curved upwards. Carapace brownish with a marbled pattern.

***Thalamita poissonii* (Audouin, 1826)**

**References**. Carpenter and De Angelis (2016a).

**Records**. MAURITANIA - Dakhlet Nouadhibou • Seagrass bed in the Baie de l’Étoile; 21.0386, -17.0236; 24.II.2023; A. Knorrn, M. Sonnewald, S. M. Moctar, and A. Freiwald leg.; hand collection; BdE-130; SMF 61113 • Old Pier; 21.0207, -17.0046; 19.II.2023; A. Knorrn, M. Sonnewald, S. M. Moctar, and A. Freiwald leg.; fish trap; BdE-103-2; SMF 61111, MAU-395.

**Identification**. Frontal margin of carapace with two very broad teeth that give the impression of continuous margin with a media incision. Last anterolateral tooth of the carapace larger than the preceding tooth. Merus of the pereopod V with one or several ventro-distal teeth.

***Carcinus maenas* (Linnaeus, 1758)**

**References**. Carpenter and De Angelis (2016a).

**Records**. MAURITANIA - Dakhlet Nouadhibou • Seagrass bed in the Baie de l’Étoile; 21.0191, -17.0063; 24.VII.2022; A. Knorrn, F. Krupp, S. M. Moctar, and A. Freiwald leg.; Beach seine; BdE-05; IMROP 15.

**Identification**. Body rather flat, finely granular, and pubescent. Frontal part of carapace with three blunt teeth. Anterolateral margin cut into five rather blunt teeth. Body colouration deep-green mottled with brown and blackish. A semicircle of whitish spots on each half of the carapace.

**Remarks**. Mauritania is probably the southernmost distribution of this species along the east Atlantic coast. Occurs in quite high densities, hiding under stones in shallow water, just like at the North Sea coasts.

***Panopeus africanus* A. Milne-Edwards, 1867**

Fig. 4E

**References**. Carpenter and De Angelis (2016a).

**Records**. MAURITANIA - Dakhlet Nouadhibou • Seagrass bed in the Baie de l’Étoile; 21.0340, -17.0261; 25.VII.2022; A. Knorrn, M. Sonnewald, S. M. Moctar, and A. Freiwald leg.; Hand collection; BdE-13; IMROP 7, MAU-236 • Rocky island; 21.0254, -17.0067; 17.II.2023; A. Knorrn and A. Freiwald leg.; hand collection; BdE-64; SMF 61108 • Pelican island; 20.7111, -16.6880; 03.III.2023; A. Knorrn, M. Sonnewald, S. M. Moctar, and A. Freiwald leg.; hand collection from a maerl and rock habitat; BdL-74; IMROP 37, SMF 61107.

**Identification**. Carapace hexagonal and rather flat, with some transverse rows of small granules on anterior half. Male abdomen elongate and narrow, with segments III–V fused, covering most of sternite four. Body reddish brown with a brownish shine. Dark brown-coloured chela with brighter tips.

***Xantho poressa* (Olivi, 1792)**

**References**. Carpenter and De Angelis (2016a).

**First record**. MAURITANIA - Dakhlet Nouadhibou • Old Pier; 21.0206, -17.0046; 02.XII.2021; M. Sonnewald, S. M. Moctar, and A. Freiwald leg.; collected underneath an old pier; CDP21-01-3; IMROP 3, SMF 58340, MAU-221.

**Identification**. Smooth and broadly oval carapace with five blunt teeth on the anterolateral margins. Frontal margin with three rounded teeth. Robust and equally sized chelae, with finely granular surfaces. Body coloration dark green with mottled yellow spots.

***Pachygrapsus transversus* (Gibbes, 1850)**

Fig. 4F

**References**. Carpenter and De Angelis (2016a).

**Records**. MAURITANIA - Dakhlet Nouadhibou • Old Pier; 21.0196, -17.0056; 15.II.2023; A. Knorrn, M. Sonnewald, S. M. Moctar, and A. Freiwald leg.; in close proximity to the old pier; BdE-42; SMF 61114.

**Identification**. Carapace slightly convex with several transverse ridges dorsally. Front with a sinuous anterior margin with a distinctly greater width than half the carapace. Lateral margin with one single tooth behind the exorbital tooth. Smooth chela without tubercles or spines. Merus of all walking legs with teeth at the posterio-distal angle. Body brownish with a green to yellowish variegated pattern.

***Dudekemus atlanticus* (Monod, 1933)**

**References**. Bocquet (1963); Muñoz et al. (2025).

**Record**. MAURITANIA - Dakhlet Nouadhibou • Baie du Lévrier; 21.0135, -16.9611; 04.XII.2021; S. M. Moctar, and A. Freiwald leg.; grab sample; BdL-21-3-2; IMROP 22, SMF 58346, MAU-217.

**Identification**. Irregular hexagonal carapace, which is wider than long with relatively small chelipeds. Pereopods II–V are strongly reduced in size. Merus, carpus and propodus of pereopods III and IV enlarged. Body overall ochre coloured with a reddish brown in the hepatic and branchial parts of the body. Pereopods III and IV have a distinct vine red at the distal part.

***Afruca tangeri* (Eydoux, 1835)**

Fig. 4G

**References**. Carpenter and De Angelis (2016a).

**Records**. MAURITANIA - Dakhlet Nouadhibou • Seagrass bed in the Baie de l’Étoile; 21.0191, -17.0063; 24.VII.2022; A. Knorrn, F. Krupp, S. M. Moctar, and A. Freiwald leg.; hand collection; BdE-5; IMROP 23 • Seagrass bed in the Bellaat lagoon; 20.6929, -16.6757; 03.III.2023; A. Knorrn, S. M. Moctar, M. Sonnewald, and A. Freiwald leg.; hand collection during low tide; BdL-69; IMROP 33, SMF 61105 • Agadir Sandy beach; 20.6106, -16.4468; 05.III.2023; M. Sonnewald and A. Knorrn leg., collected along the sandy beach of Agadir, Ile d’Arguin; IdA-14; IMROP 34.

**Identification**. Eyes slender and the orbits occupying the entire anterior margin of the front. Chelipeds in females small and equal in size. Cheliped in males unequal in size. Only one cheliped is small like that of females, the other one is very large with a chela being > 2× as long as carapace. Carapace greyish to purplish reddish. Cheliped are purplish at the base and pincers are white to partly yellowish.

***Pinnotheres pisum* (Linnaeus, 1767)**

Fig. 4H

**References**. Hayward and Ryland (2017); Monod (1956).

**Record**. MAURITANIA - Dakhlet Nouadhibou • Old Pier; 21.0197, -17.0053; 23.VII.2022; A. Knorrn, F. Krupp, S. M. Moctar, and A. Freiwald leg.; from a living *Ruditapes decussatus* bivalve from close proximity of an old pier; BdE-1; SMF 61099, MAU-241.

**Identification**. Females with translucent carapace, revealing orange-coloured internal organs. Chelae equal in length and antennas minute. Dactyl of pereopods II–V strongly curved. Body yellowish to greyish with symmetrical darker markings.

#### Mollusca

***Acanthochitona fascicularis* (Linnaeus, 1767)**

Fig. 5A

**Figure 5.**
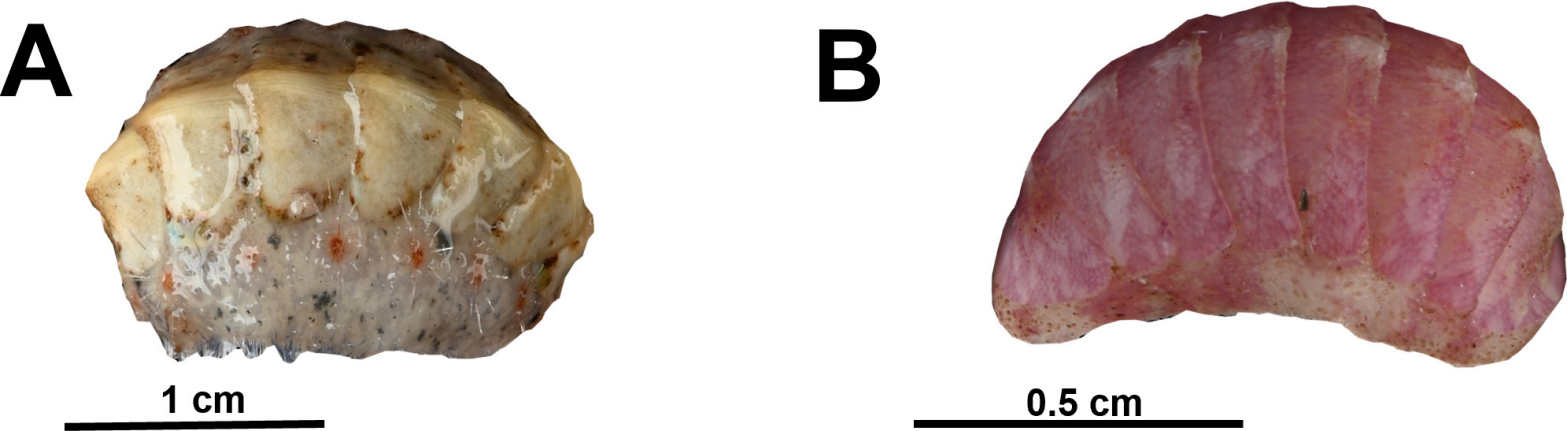
Marine polyplacophorans collected near Kijji island, northern Mauritania. **A**. *Acanthochitona fascicularis*; **B**. *Ischnochiton cessaci*. © Nicol Mahnken: A, B.

**References**. Hayward and Ryland (2017).

**First record**. MAURITANIA - Dakhlet Nouadhibou • Kijji island; 19.8227, -16.5940; 20.VI.2023; A. Knorrn and A. Freiwald leg.; ganchorra haul; K001; IMROP 147, SMF 367253, MAU-564.

**Identification**. Eight shell valves strongly arched with a rounded keel and prominent beaks. Longitudinal ridges are laterally packed with rounded granules. Gridle packed with 18 dense tufts of bristles. Very variable in colour, ranging between grey, yellowish, chestnut, olive-green, brown to some shades of blue and sometimes with streaks or a marbled pattern.

***Ischnochiton cessaci* (Rochebrune, 1881)**

Fig. 5B

**References**. Rochebrune (1881).

**Record**. MAURITANIA - Dakhlet Nouadhibou • Kijji island; 19.8227, -16.5940; 20.VI.2023; A. Knorrn and A. Freiwald leg.; ganchorra haul; K001; SMF 377905, MAU-565

**Identification**. Anterior and posterior valve are concentrically lineated in a brighter colouration than the rest of the valve. Central area of the intermediate valves striatulated with sometimes interrupted striae. Lateral areas finely undulated. No tufts of bristles present. Variable in colour, mostly pinkish to creamy white in Mauritanian waters.

***Patella rustica* Linnaeus, 1758**

**References**. Carpenter and De Angelis (2016b).

**Record**. MAURITANIA - Dakhlet Nouadhibou • Cap Blanc; 20.7709, -17.0468; 23.II.2023; A. Knorrn, F. Krupp, S. M. Moctar, and A. Freiwald leg.; hand collection; BdL-20; IMROP 138, SMF 367240.

**Identification**. Shell conical and robust, with well-defined ridges running from the apex to the margin. Apex often slightly off-centre and curved. Interior of the shell usually white with a central muscle scar. Typical grey to greenish brown in colour with radiating dark stripes.

***Patella depressa* Pennant, 1777**

Fig. 6A

**Figure 6.**
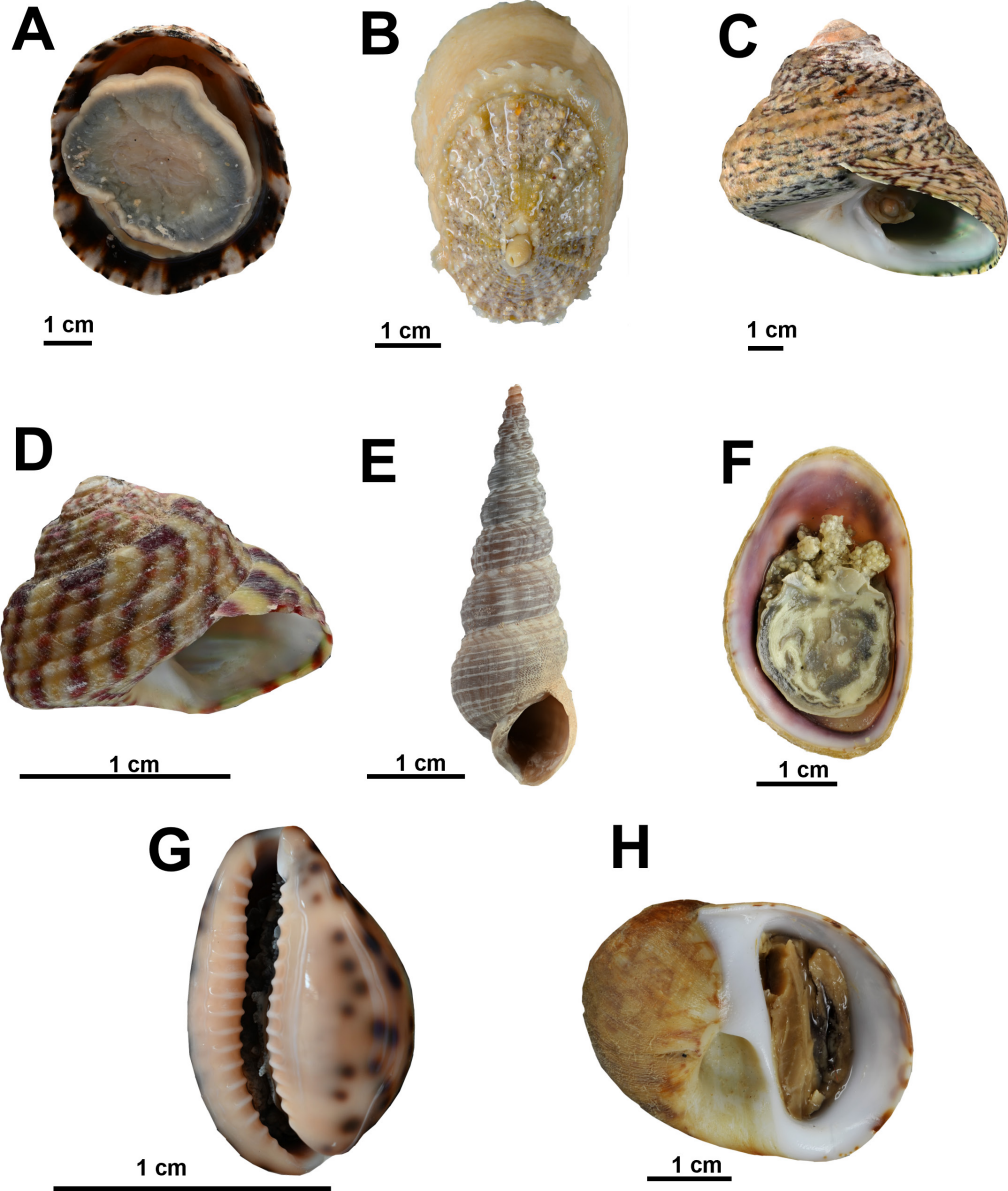
Marine gastropods from coastal habitats in northern Mauritania. **A**. *Patella depressa*; **B**. *Diodora graeca*; **C**. *Phorcus lineatus*; **D**. *Steromphala umbilicalis*; **E**. *Mesalia mesal*; **F**. *Crepidula porcellana*; **G**. *Zonaria zonaria*; **H**. *Natica fulminea*. © Nicol Mahnken.

**References**. Hayward and Ryland (2017).

**First record**. MAURITANIA - Dakhlet Nouadhibou • Rocky shore of Cansado; 20.8536, -17.028761; 26.II.2023; A. Knorrn and M. Sonnewald leg.; hand collection; BdL-34; SMF 367241, MAU-334.

**Identification**. Shell resembles a flattened cone with various fine radiating ribs. Greyish to brown shell with distinctive orange-brown rays on the inner surface. Foot dark olive to black in colouration with chalky white pallial tentacles.

***Diodora graeca* (Linnaeus, 1758)**

Fig. 6B

**References**. Graham (1988).

**First record**. MAURITANIA - Dakhlet Nouadhibou • Submerged ridges; 20.6528, -16.7316; 30.VII.2022; A. Knorrn, S. M. Moctar, F. Krupp, and A. Freiwald leg.; ganchorra haul; BdL-06-1; SMF 372467, MAU-499.

**Identification**. Shell conical with a key-hole shaped apical hole, placed ~1/3 of shell length behind the anterior end. Surface with numerous radiating ridges crossed by other radial ribs, which often have an upturned edge. Shell brownish to dirty grey. Body creamy white to a deep orange or red.

***Phorcus lineatus* (da Costa, 1778)**

Fig. 6C

**References**. Carpenter and De Angelis (2016b).

**Records**. MAURITANIA - Dakhlet Nouadhibou • Rocky island; 21.0256, -17.0071; 26.VII.2022; A. Knorrn, F. Krupp, S. M. Moctar, and A. Freiwald leg.; hand collection; BdE-20; IMROP 27, SMF 372438 • Old Pier; 21.0199, -17.0055; 24.VII.2022; A. Knorrn, S. M. Moctar, and A. Freiwald leg.; underneath boulders and around the old pier; BdE-02-1; IMROP 35, SMF 372453 • Sandstone; 21.0426, -17.0259; 24.II.2023; A. Freiwald leg.; intertidal sandstone boulders; BdE-129; IMROP 100 • Pelican island 20.7085, -16.6839, 03.III.2023, A. Knorrn, M. Sonnewald, S. M. Moctar, and A. Freiwald leg.; hand collection from a maerl and rock habitat; BdL-73; SMF 367202 • Cap Blanc; 20.7710, -17.0467; 23.II.2023; A. Knorrn, M. Sonnewald, S. M. Moctar, and A. Freiwald leg.; hand collection; BdL-22; IMROP 94, SMF 367197 • Old Stone Pier at Agadir, Ile d’Arguin; 20.6125, -16.4472; 05.III.2024; A. Knorrn, M. Sonnewald, S. M. Moctar, and A. Freiwald leg.; hand collection; IdA-06; IMROP 95, SMF 367198.

**Identification**. Solid, blunt shell, conical spire with ~5 convex whorls. Sculpture ~10 spiral cords, some of which protruding, numerous strongly prosocline growth lines, umbilical area smooth with umbilical impression. Suture shallow. Aperture rounded. Internally smooth with spiral imprint. Numerous very fine oblique dark purplish brown to green lines interrupted by other zigzag patterns or fine spiral streaks on a pale buff ground. Operculum corneous, multi-spiral.

***Steromphala umbilicalis* (da Costa, 1778)**

Fig. 6D

**References**. Carpenter and De Angelis (2016b).

**Records**. MAURITANIA - Dakhlet Nouadhibou • Maerl bed in the Baie de l’Étoile; 21.0248, -17.0071; 17.II.2023; A. Knorrn, M. Sonnewald, S. M. Moctar, and A. Freiwald leg.; hand collection; BdE-63; IMROP 133 • Old Pier; 21.0206, -17.0046; 27.II.2023; A. Knorrn, M. Sonnewald, S. M. Moctar, and A. Freiwald leg.; fish trap; BdE-142-2; IMROP 161, SMF 377915 • Old Stone Pier at Agadir, Ile d’Arguin; 20.6106, -16.4468; 05.III.2023; A. Knorrn, M. Sonnewald, S. M. Moctar, and A. Freiwald leg.; hand collection; IdA-09; IMROP 132, SMF 367235, MAU-495.

**Identification**. Shell conical and cyrtoconoid in shape with 5-6 whorls. An angular last whorl and 8-11 spiral ridges along the base of the last whorl. Umbilicus relatively large and circular. Shell cream or greenish with broad reddish or purple bands.

***Mesalia mesal* (Deshayes, 1843)**

Fig. 6E

**References**. Carpenter and De Angelis (2016b).

**Records**. MAURITANIA - Dakhlet Nouadhibou • Submerged ridges; 20.6547, -16.7260; 02.III.2023; A. Knorrn, S. M. Moctar, M. Sonnewald, and A. Freiwald leg.; ganchorra haul; BdL-53; IMROP 157, SMF 377913 • Seagrass bed in the Baie de l’Étoile; 21.0455, -17.01960; 24.VII.2022; A. Knorrn, F. Krupp, S. M. Moctar, and A. Freiwald leg.; hand collection; BdE-129; IMROP 166, MAU-341.

**Identification**. Spiral sculpture of the whorls reduced to a strong groove under the suture. Base of the columella without a spiral ridge above the recurved inner lip. Shell whitish with chestnut to fawn-coloured axial streaks.

***Mesalia opalina* (A. Adams & Reeve, 1849)**

**References**. Carpenter and De Angelis (2016b).

**Record**. MAURITANIA - Dakhlet Nouadhibou • Submerged ridges; 20.6531, -16.7267; 02.III.2023; A. Knorrn, S. M. Moctar, F. Krupp, and A. Freiwald leg.; ganchorra haul; BdL-51; SMF 377912, IMROP 156.

**Identification**. Spiral structure with a few distant ridges on the apical part of the whorls. Base of the columella with small spiral ridge, just above the recurved inner lip. Shell whitish to brownish without distinct axial streaks.

***Crepidula porcellana* Lamarck, 1801**

Fig. 6F

**References**. Carpenter and De Angelis (2016b).

**Records**. MAURITANIA - Dakhlet Nouadhibou • Maerl bed in the Baie de l’Étoile; 21.0254, -17.0073; 26.VII.2022; A. Knorrn, F. Krupp, S. M. Moctar, and A. Freiwald leg.; hand collection; BdE-20-1; SMF 372432, MAU-261 • Seagrass bed in the Baie de l’Étoile; 21.0383, -17.0260; 24.II.2023; A. Knorrn, M. Sonnewald, S. M. Moctar, and A. Freiwald leg.; hand collection; BdE-131; IMROP 120, SMF 367224 • Old Pier; 21.0207, -17.0046; 19.II.2023; A. Knorrn, M. Sonnewald, S. M. Moctar, and A. Freiwald leg.; fish trap; BdE-103-2; SMF 367219 • Submerged ridges; 20.6531, -16.7267; 02.III.2023; A. Knorrn, S. M. Moctar, M. Sonnewald, and A. Freiwald leg.; ganchorra haul; BdL-51; SMF 367230 • Pelican island; 20.7111, -16.6880; 03.III.2023; A. Knorrn, M. Sonnewald, S. M. Moctar, and A. Freiwald leg.; hand collection; BdL-74; IMROP 118, SMF 367222 • Near Old Stone Pier at Agadir, Ile d’Arguin; 20.6106, -16.4468; 05.III.2023; A. Knorrn, M. Sonnewald, S. M. Moctar, and A. Freiwald leg.; hand collection; IdA-09; IMROP 127.

**Identification**. Oval and flattened shell, resembling a slipper. Interior smooth and white, with a prominent septum forming a shelf-like structure. Exterior surface smooth with fine growth lines. Shell white to pale brown, often with darker blotches.

***Zonaria zonaria* (Gmelin, 1791)**

Fig. 6G

**References**. Carpenter and De Angelis (2016b).

**Record**. MAURITANIA - Dakhlet Nouadhibou • Submerged ridges; 20.6826, -16.7168; 04.III.2023; A. Knorrn, S. M. Moctar, M. Sonnewald, and A. Freiwald leg.; ganchorra haul; BdL-86; SMF 367205, MAU-482.

**Identification**. Shell ovate and spire short and concealed under the body whorl. Both lips with raised transverse ridges or teeth, the outer one thickened and incurved. Inner lip with a shallow longitudinal furrow situated towards the frontal end. Surface highly polished, smooth, and lateral shell margins with several dark spots. Ventral side of shell cream to pale greyish.

***Natica fulminea* (Gmelin, 1791)**

Fig. 6H

**References**. Carpenter and De Angelis (2016b).

**Records**. MAURITANIA - Dakhlet Nouadhibou • Kijji island; 19.8227, -16.5940; 20.VI.2023; A. Knorrn and A. Freiwald leg.; ganchorra haul; K001; SMF 367193 • Submerged ridges; 20.6528, -16.7316; 30.VII.2022; A. Knorrn, S. M. Moctar, F. Krupp, and A. Freiwald leg.; ganchorra haul; BdL-06-1; SMF 372433, MAU-266 • Seagrass bed in the Baie de l’Étoile; 21.0217, -17.0065; 16.II.2023; A. Knorrn, M. Sonnewald, S. M. Moctar, and A. Freiwald leg.; hand collection; BdE-54; IMROP 92, SMF 367194.

**Identification**. Shell globose and approximately as long as wide with a short spire. Moderately convex whorls and a very large body whorl that is slightly flattened at the suture. Umbilicus widely open without a funicle. Outside of shell creamy white with a variable reddish to brownish colour pattern typically composed of angular zigzag lines. Interior and the inner lip callus are white.

***Bolinus cornutus* (Linnaeus, 1758)**

Fig. 7A

**Figure 7.**
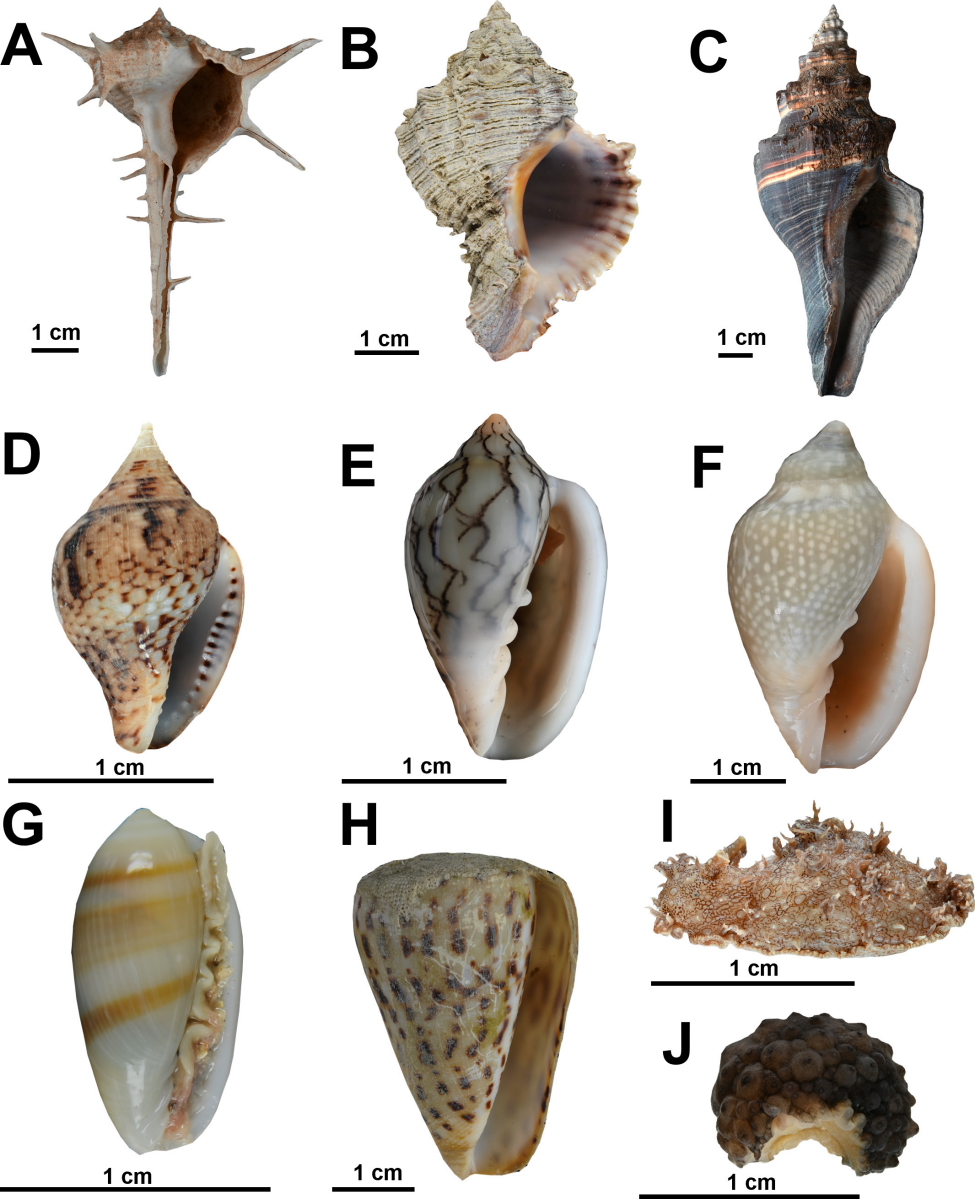
Marine gastropods from coastal habitats in northern Mauritania. **A**. *Bolinus cornutus*; **B**. *Hexaplex rosarium*; **C**. *Pugilina morio*; **D**. *Columbella adansoni*; **E**. *Marginella cleryi*; **F**. *Marginella glabella*; **G**. *Volvarina ampelusica*; **H**. *Conus byssinus*; **I**. *Bursatella leachii*; **J**. *Onchidella celtica*. © Nicol Mahnken: B–J, © Kristina Hopf: A.

**References**. Carpenter and De Angelis (2016b).

**Record**. MAURITANIA - Dakhlet Nouadhibou • Seagrass bed in the Baie de l’Étoile; 21.0449, -17.0224; 31.I.2024; A. Knorrn, M. Sonnewald, S. M. Moctar, and A. Freiwald leg.; hand collection; BdE-191; IMROP 148.

**Identification**. Shell large and club shaped, with a very narrow and long siphonal canal. Outer surface of the shell with several varices. Outer part of shell yellowish to beige to tan, often with three darker spiral bands. The aperture is glossy and whitish, often with a paler orange part around the outer lip edge and the columellar callus.

***Hexaplex rosarium* (Röding, 1798)**

Fig. 7B

**References**. Carpenter and De Angelis (2016b).

**First records**. MAURITANIA - Dakhlet Nouadhibou • Maerl bed in the Baie de l’Étoile; 21.0256, -17.0071; 26.VII.2022; A. Knorrn, F. Krupp, S. M. Moctar, and A. Freiwald leg.; hand collection; BdE-20; IMROP 20 • Old Pier; 21.0200, -17.0050; 24.VII.2022; A. Knorrn, S. M. Moctar, and A. Freiwald leg.; hand collection; BdE-06; IMROP 45, MAU-259.

**Identification**. Shell globose to ovate in shape with a relatively low and pointed spire. Body whorl large and globose with 6-8 knobby axial varices. Outer part of shell whitish creamy to fawn with ≤ 3 brown spiral bands. Aperture glossy white within and the inner lip is bright reddish to pink in colouration.

***Stramonita haemastoma* (Linnaeus, 1767)**

**References**. Carpenter and De Angelis (2016b).

**First record**. MAURITANIA - Dakhlet Nouadhibou • Rocky shore of Cansado; 20.8536, -17.0287; 26.II.2023; A. Knorrn and M. Sonnewald leg.; hand collection; BdL-34; IMROP 32, SMF 372446, MAU-336.

**Identification**. Shell relatively thick and almost biconical in shape. Surface with a variable arrangement of fine incised spiral lines. Outer lip crenulated with numerous spiral grooves. Outside of shell creamy white to dirty grey with areas of brighter and darker colouration. Aperture bright orange to salmon-like colouration often tinged with a darker brown on the grooves around the outer lip margins.

***Pugilina morio* (Linnaeus, 1758)**

Fig. 7C

**References**. Carpenter and De Angelis (2016b).

**Records**. MAURITANIA - Dakhlet Nouadhibou • Seagrass bed in the Baie de l’Étoile; 21.0191, -17.0063; 24.VII.2022; A. Knorrn, F. Krupp, S. M. Moctar, and A. Freiwald leg.; hand collection; BdE-05; IMROP 29, SMF 372464, MAU-263 • Maerl bed in the Baie de l’Étoile; 21.0202, -17.0046; 20.II.2023; A. Knorrn, M. Sonnewald, S. M. Moctar, and A. Freiwald leg.; hand collection; BdE-117; SMF 367156 • Pelican island; 20.7111, -16.6880; 03.III.2023; A. Knorrn, M. Sonnewald, S. M. Moctar, and A. Freiwald leg.; hand collection; BdL-74; IMROP 158 • Old Pier; 21.0207, -17.0046; 19.II.2023; A. Knorrn, M. Sonnewald, S. M. Moctar, and A. Freiwald leg.; fish trap; BdE-103-2; IMROP 164.

**Identification**. Shell more or less fusiform with a rather high spire and expanded body whorl. Surface of shell with a single row of blunt spines along the angular shoulder and many low spiral threads all along the shell. Aperture large and subquadrate narrowing into the siphonal canal. Shell coloured in a dark brown to black with one or several contrasting spiral band in a brighter brown. Periostracum relatively thick and dark brownish, strong enough to hide the colouration of the shell.

***Tritia pfeifferi* (R. A. Philippi, 1844)**

**References**. Adam and Knudsen (1984).

**Records**. MAURITANIA - Dakhlet Nouadhibou • Pelican island; 20.7111, -16.6880; 03.III.2023; A. Knorrn, M. Sonnewald, S. M. Moctar, and A. Freiwald leg.; hand collection; BdL-74; SMF 377917 • Seagrass bed in the Baie de l’Étoile; 21.0191, -17.0063; 24.VII.2022; A. Knorrn, F. Krupp, S. M. Moctar, and A. Freiwald leg.; hand collection; BdE-05; IMROP 154.

**Identification**. Solid, elevated shell, conical spire with ~5 weakly convex whorls with weak shoulder area, apex finely rounded, apical angle 45 degrees. External surface smooth with numerous prosocline growth lines, umbilical area covered by callus. Suture shallow impressed. Aperture piriform, lip thickened, denticles on inside external lip; wide twisted siphonal channel. Internally smooth. Background colour white, most of external shell covered by dark brown blotches, axial bands leaving most of the callus and internal area white.

***Columbella adansoni* Menke, 1853**

Fig. 7D

**References**. Hernández et al. (2011).

**Record**. MAURITANIA - Dakhlet Nouadhibou • Old Pier; 21.0207, -17.0046; 19.II.2023; A. Knorrn, M. Sonnewald, S. M. Moctar, and A. Freiwald leg.; fish trap; BdE-103-2; SMF 377910, IMROP 155, MAU-503.

**Identification**. Solid, elevated shell, conical spire with ~5 flattened whorls with corrugated shoulder area, apex rounded. External surface largely smooth with numerous prosocline growth lines. Suture shallow. Aperture flexuous, strongly protruding lip, denticles on inside external lip, wide twisted siphonal channel, internally smooth. Background colour white, most of external shell covered by pale to dark brown blotches, axial bands leaving only internal area white.

***Mitrella broderipii* (G. B. Sowerby I, 1844)**

**References**. Hernández et al. (2011).

**Records**. MAURITANIA - Dakhlet Nouadhibou • Maerl bed in the Baie de l’Étoile; 21.0184, -17.0030; 20.II.2023; A. Knorrn, F. Krupp, S. M. Moctar, and A. Freiwald leg.; hand collection; BdE-119; SMF 377909

**Identification**. Solid, highly elevated shell, conical spire with ~6 convex whorls, apex rounded. External surface largely smooth with numerous slightly prosocline growth lines, columellar area with spiral grooves. Suture shallow, impressed. Aperture elongated piriform, very weak fine denticles on inside external lip, wide twisted siphonal channel, columella straightened, slightly s-shaped, internally smooth. Background creamy white, most of external shell covered by regular axial brown bands that may be crossed by broad spiral white, pale, or dark brown bands.

***Aplus assimilis* (Reeve, 1846)**

**References**. Aissaoui et al. (2016).

**Records**. MAURITANIA - Dakhlet Nouadhibou • Old Pier; 21.0207, -17.0046; 19.II.2023; A. Knorrn, M. Sonnewald, S. M. Moctar, and A. Freiwald leg.; fish trap; BdE-103-2; IMROP 162, SMF 377916 • Rocky island; 21.0254, -17.0067; 17.II.2023; A. Knorrn, M. Sonnewald, S. M. Moctar, and A. Freiwald leg.; hand collection; BdE-64; IMROP 159, SMF 377914.

**Identification**. Solid, elevated shell, conical spire with ~6 flattened whorls, apex finely rounded. Sculpture with coarse spiral cords and flexuous ribs of equal strength. Suture shallow, impressed. Aperture elongated piriform, spiral notches on inside external lip, wide twisted siphonal channel, columella straightened, slightly s-shaped. Internally with imprint of external spiral sculpture. Background creamy white to dark brown, pale band at suture and near base.

***Cymbium marmoratum* Link, 1807**

**References**. Carpenter and De Angelis (2016b).

**Record**. MAURITANIA - Dakhlet Nouadhibou • Fishermen from the Baie de l’Étoile; 21.0198, -17.0028; 09.III.2023; A. Knorrn, S. M. Moctar, and A. Freiwald leg.; in front of the BdE opening; gill net of local fishermen (tissue sample); Mau-335 • Rocky island; 21.0251, -17.0070; 03.II.2024; M. Sonnewald leg.; small specimen from a fish trap in close proximity to seagrass beds; BdE-258; SMF 377906.

**Identification**. Shell moderately large (≤20 cm) and solid with an elongated to ovate but somewhat inflated shape. A large V-shaped channel developed between shoulder ridge and spire whorls. The shell is marbled reddish brown and white on a pale brown base.

**Remarks**. Intensively fished by local fishermen at the Baie de l’Étoile, despite the formal designation as a marine protected area. Should be considered to enter the IUCN Red List of threatened species.

***Marginella cleryi* Petit de la Saussaye, 1836**

Fig. 7E

**References**. Cossignani (2006).

**Records**. MAURITANIA - Dakhlet Nouadhibou • Submerged ridges; 20.6547, -16.7260; 02.III.2023; A. Knorrn, S. M. Moctar, M. Sonnewald, and A. Freiwald leg.; ganchorra haul; BdL-53; IMROP 83 • Seagrass bed in the Baie de l’Étoile; 21.0247, -17.0065; 19.II.2023; A. Knorrn, M. Sonnewald, S. M. Moctar, and A. Freiwald leg.; hand collection; BdE-109; SMF 367184, MAU-481.

**Identification**. Solid, elevated shell, conical spire with ~4 slightly convex whorls, apex rounded. Surface smooth, polished. Suture smeared. Aperture elongate, smooth thickened external lip, wide twisted siphonal channel, columella straightened, slightly concave, four spiral folds of which the lower three are strongly oblique, internally smooth. Background bluish white, fine dark brown to black axial lines in complex zig-zag pattern, frequently bifurcating or merging.

***Marginella glabella* (Linnaeus, 1758)**

Fig. 7F

**References**. Carpenter and De Angelis (2016b).

**Record**. MAURITANIA - Dakhlet Nouadhibou • Seagrass bed in the Baie de l’Étoile; 21.0367, -17.0258; 24.II.2023; A. Knorrn, M. Sonnewald, S. M. Moctar, and A. Freiwald leg.; hand collection; BdE-133; IMROP 85, SMF 367187.

**Identification**. Shell elongated, oval in shape with a smooth and highly polished surface. Aperture elongated with a relatively short anterior siphonal canal. Outer lip thickened and reflected interiorly. Shell creamy brown coloured with several weakly defined white spots that are irregularly distributed about the shell. Animal purplish to reddish coloured with fine pale-coloured streaks.

***Volvarina ampelusica* Monterosato, 1906**

Fig. 7G

**References**. Hernández et al. (2011).

**Records**. MAURITANIA - Dakhlet Nouadhibou • Submerged ridges; 20.6528, -16.7316; 30.VII.2022; A. Knorrn, S. M. Moctar, F. Krupp, and A. Freiwald leg.; ganchorra haul; BdL-06-1; IMROP 31, SMF 372443 • Seagrass bed in the Baie de l’Étoile; 21.0224, -17.0062; 16.II.2023; A. Knorrn, M. Sonnewald, S. M. Moctar, and A. Freiwald leg.; hand collection from a sand seabed in close proxymity to a *Cymodocea nodosa* bed; BdE-55; IMROP 108.

**Identification**. Solid, convolved shell, small blunt spire with ~4 whorls, apex rounded. Surface smooth, polished. Suture smeared. Aperture elongated and curved, inside external lip with denticles, wide twisted siphonal channel, columella straightened, slightly concave, three to four strongly oblique folds, internally smooth. Background white to pale brown, spiral brown bands.

***Persicula cingulata* (Dillwyn, 1817)**

**References**. Rolán (2005).

**Records**. MAURITANIA - Dakhlet Nouadhibou • Kijji island; 19.8227, -16.5940; 20.VI.2023; A. Knorrn and A. Freiwald leg.; ganchorra haul; K001; SMF 367213 • Seagrass bed in the Baie de l’Étoile; 21.0367, -17.0258; 24.II.2023; A. Knorrn, M. Sonnewald, S. M. Moctar, and A. Freiwald leg.; hand collection; BdE-133; IMROP 160.

**Identification**. Solid, convolved shell, tiny blunt spire with sutural groove. Surface smooth, polished. Aperture elongated and strongly curved, lip externally embossed, also at siphonal channel, denticles on inside external lip and on columella, large calloused notch on apical end of parietal margin, wide siphonal channel, columella and parietal area convex. Shell internally smooth. Background white, external spiral brown colour bands, occasionally merging towards the lip.

***Conus byssinus* (Röding, 1798)**

Fig. 7H

**References**. Filmer (2001).

**Records**. MAURITANIA - Dakhlet Nouadhibou • Maerl bed in the Baie de l’Étoile; 21.0256, -17.0071; 24.II.2022 A. Knorrn, F. Krupp, S. M. Moctar, and A. Freiwald leg.; hand collection; BdE-20-1; IMROP 19 • Seagrass bed in the Baie de l’Étoile; 21.0383, -17.0260; 24.II.2023 A. Knorrn, S. M. Moctar, and A. Freiwald leg.; hand collection; BdE-131; SMF 372476.

**Identification**. Solid, convolved shell, broad blunt spire, conical, five to six whorls. Suture deeply impressed. Ultimate whorl conical, slightly convex with blunt keeled shoulder. Surface smooth, with fine, nearly orthocline, growth lines. Aperture elongate, very weakly curved, lip thin, straight wide siphonal end, internally smooth. Background cream white, externally with broad pale brown spiral bands and dark brown or black interrupted patterns of notches variable in size, but both spirally and axially aligned.

***Bulla striata* Bruguière, 1792**

**References**. Hernández et al. (2011).

**Records**. MAURITANIA - Dakhlet Nouadhibou • Seagrass bed in the Baie de l’Étoile; 21.0390, -17.0235; 24.II.2023; A. Knorrn, S. M. Moctar, M. Sonnewald, and A. Freiwald leg.; Beach seine; BdE-128; IMROP 145 • Pelican island; 20.7111, -16.6880; 03.III.2023; A. Knorrn, M. Sonnewald, S. M. Moctar, and A. Freiwald leg.; hand collection; BdL-74; IMROP 144, SMF 367249.

**Identification**. Thin, convolved shell, oblong, oval, spire sunken, surface smooth, with fine, nearly orthocline, growth lines. Aperture elongate piriform, narrow adapically, wide abapically, lip thin, straightened upper half. Demarcated white callus on columella and parietal area. Diameter ≤ 10 mm. Background cream white, externally pale brown with dark brown, axially aligned blotches.

***Bursatella leachii* Blainville, 1817**

Fig. 7I

**References**. Hayward and Ryland (2017).

**Records**. MAURITANIA - Dakhlet Nouadhibou • Submerged ridges; 20.6547, -16.7260; 02.III.2023; A. Knorrn, S. M. Moctar, M. Sonnewald, and A. Freiwald leg.; ganchorra haul; BdL-53; IMROP 34, SMF 367243, IMROP 140 • Seagrass bed in the Baie de l’Étoile; 21.0389, -17.0250; 31.I.2024; A. Knorrn, M. Sonnewald, S. M. Moctar, and A. Freiwald leg.; hand collection; BdE-201; IMROP 151.

**Identification**. The body is oval and flattened, covered with numerous fleshy papillae and small, branched papillae. Anteriorly, two short rhinophores and large, lobed oral tentacles are prominent. This species also features a broad foot, which is often fringed. A mottled coloration of green, brown, and blue eye-spots, often with distinctive blue-green tinge.

***Onchidella celtica* (Audouin & Milne-Edwards, 1832)**

Fig. 7J

**References**. Hayward and Ryland (2017).

**Record**. MAURITANIA - Dakhlet Nouadhibou • Rocky island; 21.0256, -17.0071; 26.VII.2022; A. Knorrn, F. Krupp, S. M. Moctar, and A. Freiwald leg.; hand collection; BdE-20; IMROP 25, SMF 372436, MAU-258.

**Identification**. Mantle covered with large and well-spaced tubercles. Body dark olive green to black in colour and fleshy, oval in shape. Two short and thick tentacles are visible anteriorly.

***Siphonaria pectinata* (Linnaeus, 1758)**

**References**. Hayward and Ryland (2017).

**Records**. MAURITANIA - Dakhlet Nouadhibou • Rocky island; 21.0256, -17.0071; 26.VII.2022; A. Knorrn, F. Krupp, S. M. Moctar, and A. Freiwald leg.; hand collection; BdE-20-1; IMROP 30, SMF 372442, MAU-262 • Old Pier 21.0197, -17.0053; 23.VII.2022; A. Knorrn, F. Krupp, S. M. Moctar, and A. Freiwald leg.; hand collection; BdE-1; IMROP 149 • Old Stone Pier at Agadir, Ile d’Arguin; 20.6106, -16.4468; 05.III.2023; A. Knorrn, M. Sonnewald, S. M. Moctar, and A. Freiwald leg.; hand collection; IdA-09; IMROP 103, SMF 367206.

**Identification**. Body is cap-shaped and low in profile with a broad base. Surface is covered with fine radial ribs, and the apex is slightly off-centre. Interior smooth and white, with a distinct muscle scar. The shell is grey with darker radial lines.

***Lembulus bicuspidatus* (A. Gould, 1845)**

**References**. von Cosel and Gofas (2019).

**Record**. MAURITANIA - Dakhlet Nouadhibou • Baie du Lévrier; 21.0135, -16.9611; 04.XII.2021; S. M. Moctar, and A. Freiwald leg.; grab sample; BdL21-03-2; IMROP 7, SMF 366104.

**Identification**. Shell bicuspid and inflated. Anterior margin rounded and posterior end bicuspid. Surface with numerous slightly irregular striae. Escutcheon very long and broad. Ligament restricted well behind the beaks. Chevrons very narrow and ≤ 8 in regular specimens. Valve white interiorly and exteriorly.

***Acar olivercoseli* M. Huber, 2010**

Fig. 8A

**Figure 8.**
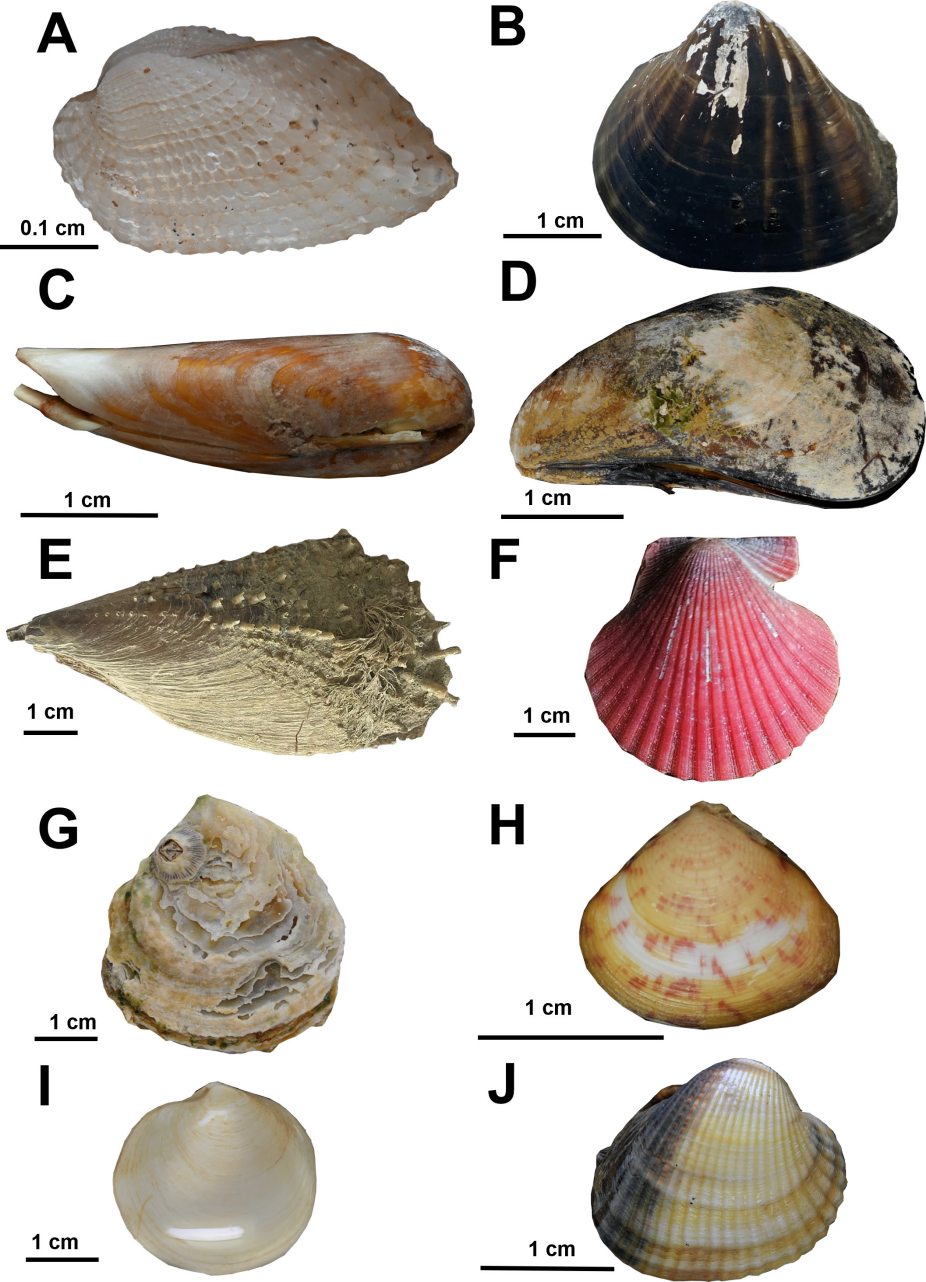
Marine bivalves from coastal habitats in northern Mauritania. **A**. *Acar olivercoseli*; **B**. *Senilia senilis*; **C**. *Leiosolenus aristatus*; **D**. *Perna perna*; **E**. *Atrina chautardi*; **F**. *Aequipecten flabellum*; **G**. *Ostrea stentina*; **H**. *Crassatina guineensis*; **I**. *Lucinoma borealis*; **J**. *Cerastoderma edule*. © Nicol Mahnken: A–D, G–J, © Kai Pfennings: F, © Alexander Knorrn: E.

**References**. von Cosel and Gofas (2019).

**First record**. MAURITANIA - Dakhlet Nouadhibou • Kijji island; 19.8227, -16.5940; 20.06.2023; A. Knorrn and A. Freiwald leg.; ganchorra sample; K001; SMF 367180.

**Identification**. Shell subrectangular or subtrapezoidal in shape and slightly inflated. Surface with ≤ 30 broad commarginal ridged crossed by 25-35 radial ribs. Shell dirty creamy on the outside and whitish inside.

***Senilia senilis* (Linnaeus, 1758)**

Fig. 8B

**References**. von Cosel and Gofas (2019).

**Records**. MAURITANIA - Dakhlet Nouadhibou • Kijji island; 19.8249, -16.5954; 20.VI.2023; A. Knorrn., E. Serrao and A. Freiwald; collected during snorkelling along seagrass beds; K003; MAU-447 • Agadir Sandy beach; 20.6106, -16.4468; 05.III.2023; M. Sonnewald and A. Knorrn, collected during snorkelling; IdA-09; IMROP 63, SMF 367162.

**Identification**. Shell approximately as high as long and subrhomboidal to triangular with strongly projecting submedian umbones. Outer sculpture with 12 (10-15) ribs with narrow interspaces. Hinge plate very broad with ≤ 15 anterior and 16 posterior teeth. Outside of shell whitish, covered by an olive green to dark brown or black periostracum.

***Leiosolenus aristatus* (Dillwyn, 1817)**

Fig. 8C

**References**. von Cosel and Gofas (2019).

**Record**. MAURITANIA - Dakhlet Nouadhibou • Rocky island; 21.0254, -17.0067; 17.II.2023; A. Knorrn and A. Freiwald leg.; hand collection; BdE-64; IMROP 76, SMF 367175.

**Identification**. Elongated shell, subcylindrical, and posteriorly narrowed. Each shell ends in a pointed tip. A smooth hinge line without teeth or crenulations. Shell ochre to whitish. Periostracum brownish and encrusted with a greyish calcareous layer which projects beyond the posterior margin and ends at the pointed tips.

***Perna perna* (Linnaeus, 1758)**

Fig. 8D

**References**. von Cosel and Gofas (2019).

**Records**. MAURITANIA - Dakhlet Nouadhibou • Rocky island; 21.0259, -17.0070; 26.VII.2022; A. Knorrn, F. Krupp, S. M. Moctar, and A. Freiwald leg.; from littoral rocks; BdE-20-3; IMROP 40, SMF 372458 • Cap Blanc; 20.7709, -17.0468; 23.II.2023; A. Knorrn, S. M. Moctar, M. Sonnewald, and A. Freiwald leg.; hand collection from rocky shoreline; BdL-20; IMROP 69, SMF 367168.

**Identification**. Shell very variable in shape, but roughly trigonal-ovate and elongated. Posterior retractor scars are widely separated in two different groups. No anterior adductor scars. Ligamental ridge finely pitted. Shell lightly purplish to brownish underneath the yellowish, greenish, or brownish periostracum.

***Atrina chautardi* (Nicklès, 1953)**

Fig. 8E

**References**. von Cosel and Gofas (2019).

**Record**. MAURITANIA - Dakhlet Nouadhibou • Seagrass bed in the Baie de l’Étoile; 21.0248, -17.0071; 17.II.2023; A. Knorrn, M. Sonnewald, S. M. Moctar, and A. Freiwald leg.; hand collection of dead shell at the seagrass bed in the Baie de l’Étoile; BdE-63; SMF 377920.

**Identification**. Shell quite variable in outline and structure, but generally elongated and ham-shaped. Shell very thin and fragile. Outer surface with 12-20 or more irregular ribs with several scaly spines. Eight to ten clearly defined ribs close to the dorsal margin. Outside of shell horn to dark brownish, somewhat greenish brown.

***Aequipecten flabellum* (Gmelin, 1791)**

Fig. 8F

**References**. von Cosel and Gofas (2019).

**Record**. MAURITANIA - Inchiri • Sandy beach at Blaouakh; 18.5187, -16.0751; 23.I.2024; A. Knorrn, K. Pfennings, and K. Hopf leg.; hand collection; B2.

**Identification**. Both valves convex with the right valve slightly more inflated than the left. Ears well developed and approx. the same size. Colouration very variable but mostly bright vermilion to pink, brownish red to purple. Interior shiny white with brownish exterior colour showing through near the margin.

***Magallana gigas* (Thunberg, 1793)**

**References**. von Cosel and Gofas (2019).

**Record**. MAURITANIA - Dakhlet Nouadhibou • Seagrass bed in the Baie de l’Étoile from an abandoned aquaculture; 21.0376, -17.0257; 24.II.2023; A. Knorrn, M. Sonnewald, S. M. Moctar, and A. Freiwald leg.; hand collection; BdE-134; IMROP 56; SMF 372479.

**Identification**. Shell elongated and ovate in outline. Outside of shell dirty white to creamy yellowish. Interior whitish and shiny, sometimes with chalky white patches. Posterior adductor scar pale.

***Ostrea stentina* Payraudeau, 1826**

Fig. 8G

**References**. von Cosel and Gofas (2019).

**Record**. MAURITANIA - Dakhlet Nouadhibou • Rocky island; 21.0256, -17.0071; 26.VII.2022; A. Knorrn, F. Krupp, S. M. Moctar, and A. Freiwald leg.; hand collection; BdE-20-1; IMROP 26, SMF 372437, MAU-252.

**Identification**. Shell very variable in shape but mostly higher than long, rounded, or irregularly oval. Outer surface of left valve with ~12 radial ribs or folds disappearing towards the dorsal half of the shell. Outside of shell greyish to brownish in colouration. Interior of shell tinged greenish, bluish grey to purplish brown.

***Crassatina alba* Cosel, 1995**

**References**. von Cosel and Gofas (2019).

**Record**. MAURITANIA - Dakhlet Nouadhibou • Submerged ridges; 20.6528, -16.7316; 30.VII.2022; A. Knorrn, S. M. Moctar, F. Krupp, and A. Freiwald leg.; ganchorra haul; BdL-06-1; IMROP 152, SMF 377908, MAU-249.

**Identification**. Umbonal region often eroded and the inner margin finely crenulated. Interior whitish. Shell rounded to trigonal in shape and slightly longer than high. Outer surface with numerous regular ridges which disappear in posterior parts of the shell. Shell whitish and covered by a pale greenish brown to darker brown periostracum.

***Crassatina guineensis* Cosel & Gofas, 2018**

Fig. 8H

**References**. von Cosel and Gofas (2019).

**Record**. MAURITANIA - Dakhlet Nouadhibou • Submerged ridges; 20.6547, -16.7260; 02.III.2023; A. Knorrn, S. M. Moctar, M. Sonnewald, and A. Freiwald leg.; ganchorra haul; BdL-53; SMF 367204, MAU-453.

**Identification**. Shell somewhat triangular in shape with a strong hinge plate. Surface with several slightly irregular ridges which disappear at the posterior parts of the shell. Shell whitish to pinkish white with pinkish to red blotches and zigzag markings. Periostracum pale yellowish brown to dark brown and usually persistent around the whole shell. Interior white, to pinkish white or lightly brownish.

***Lucinoma borealis* (Linnaeus, 1767)**

Fig. 8I

**References**. von Cosel and Gofas (2019).

**Record**. MAURITANIA - Dakhlet Nouadhibou • Baie du Lévrier; 21.0135, -16.9611; 04.XII.2021; S. M. Moctar, and A. Freiwald leg.; grab sample; BdL21-05-2; IMROP 167, SMF 377919, MAU-213.

**Identification**. Shell subcircular and rather inflated. Surface with numerous fine irregular commarginal ridges or lamellae. The posterior area is delimited by a very faint radial depression. Exterior and interior parts of the shell white. Very thin periostracum and greyish yellow to pale brown and often partly eroded.

***Cerastoderma edule* (Linnaeus, 1758)**

Fig. 8J

**References**. von Cosel and Gofas (2019).

**Records**. MAURITANIA - Dakhlet Nouadhibou • Seagrass bed in the Baie de l’Étoile; 21.0426, -17.0259; 24.II.2023; A. Knorrn, M. Sonnewald, S. M. Moctar, and A. Freiwald leg.; hand collection; BdE-129; SMF 367189 • Submerged ridges; 20.6531, -16.7267; 02.III.2023; A. Knorrn, S. M. Moctar, M. Sonnewald, and A. Freiwald leg.; ganchorra haul; BdL-51; IMROP 88.

**Identification**. Shell strongly inflated and a bit longer than high. Outer surface consists of 22–28 radial ribs that are rounded and flattened at the median part of both valves. Outer part of the shell dirty whitish to brownish yellowish. Interior whitish and becomes chestnut coloured in a posterior direction.

***Papillicardium papillosum* (Poli, 1791)**

Fig. 9A

**Figure 9.**
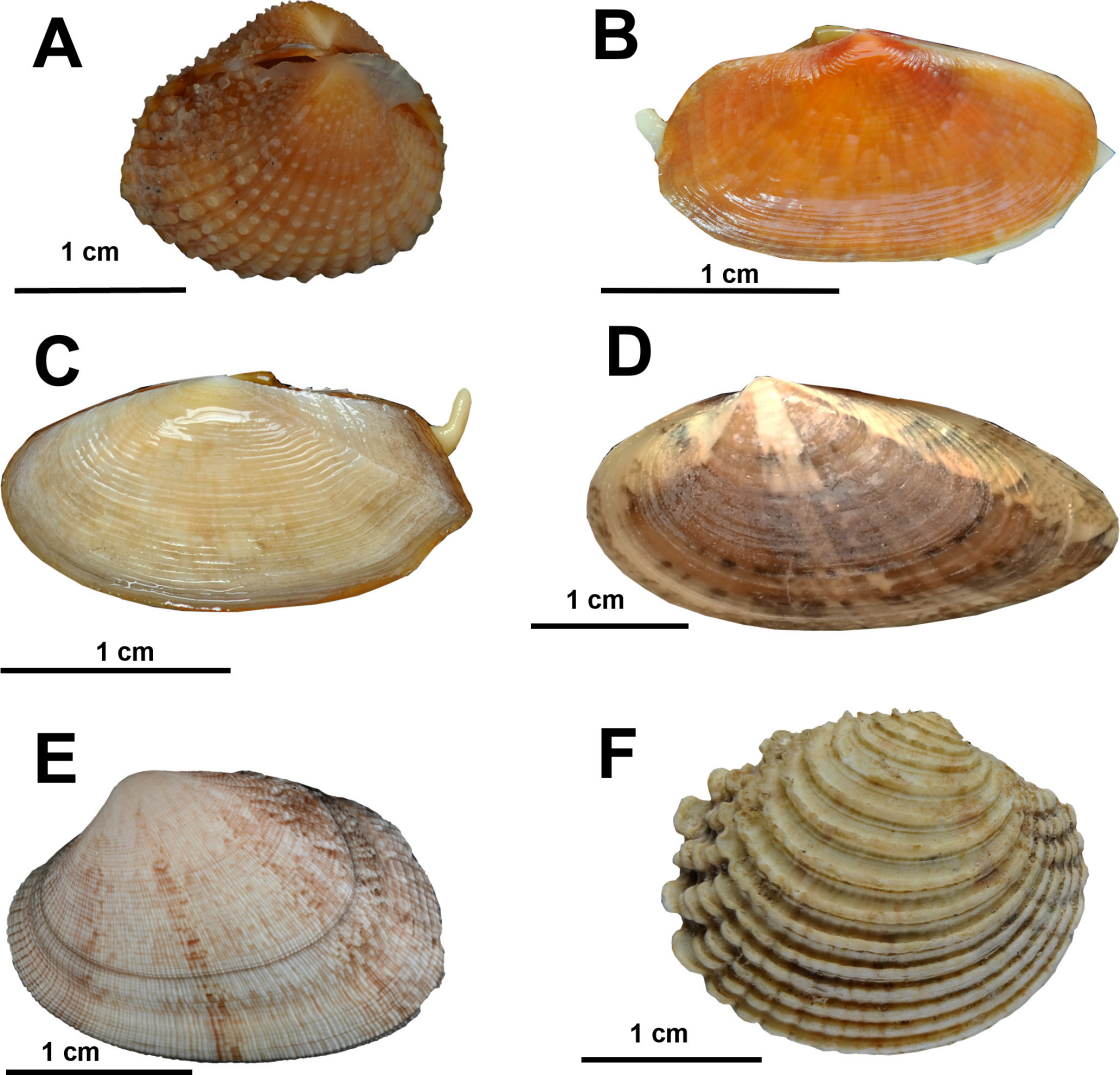
Marine bivalves from coastal habitats in northern Mauritania. **A**. *Papillicardium papillosum*; **B**. *Gari jousseaumeana*; **C**. *Gari depressa*; **D**. *Callista floridella*; **E**. *Venerupis corrugata*; **F**. *Venus verrucosa*. © Nicol Mahnken: A–F.

**References**. von Cosel and Gofas (2019).

**Records**. MAURITANIA - Dakhlet Nouadhibou • Kijji island; 19.8227, -16.5940; 20.06.2023; A. Knorrn and A. Freiwald leg.; ganchorra sample; K001; IMROP 150, Mau-457 • Submerged ridges; 20.6547, -16.7260; 02.III.2023; A. Knorrn, S. M. Moctar, M. Sonnewald, and A. Freiwald leg.; ganchorra haul; BdL-53; IMROP 89, SMF 367190, MAU-450.

**Identification**. Shell subcircular to oval and longer than high. Surface with 24–26 strong and rather flat radial ribs bearing bead-like nodules. Interspace narrow with dense transverse grooves. Outside of shell whitish to pale pinkish with irregular commarginal reddish brown streaks or markings. Interior white to reddish brown with pinkish to purple near the posterior margin.

***Gastrana matadoa* (Gmelin, 1791)**

**References**. von Cosel and Gofas (2019).

**Record**. MAURITANIA - Dakhlet Nouadhibou • Seagrass bed in the Baie de l’Étoile; 21.0224, -17.0062; 16.II.2023; A. Knorrn, M. Sonnewald, S. M. Moctar, and A. Freiwald leg.; push-core sample from a sand channel separating two seagrass beds; BdE-55; SMF 367183.

**Identification**. Shell irregular in shape but generally suboval and rather inflated. Anterior margin well rounded. Surface of shell with coarse and irregular commarginal lamellae and very fine radial depressions that end in a slight sinuosity at the posterio-ventral margin. Shell whitish to pale yellowish on the outside, whitish to pale orange posteriorly, and yellowish around the umbonal part on the interior. Periostracum brownish and only present at marginal parts of the shell.

***Huberimactra inconstans* (Cosel, 1995)**

**References**. von Cosel and Gofas (2019).

**Record**. MAURITANIA - Dakhlet Nouadhibou • Submerged ridges; 20.6547, -16.7260; 02.III.2023; A. Knorrn, S. M. Moctar, M. Sonnewald, and A. Freiwald leg.; ganchorra haul; BdL-53; SMF 377911.

**Identification**. Shell more or less subtriangular and can be very inflated. Anterior margin broadly rounded. Surface is smooth with fine irregular growth lines. Shell interiorly and exteriorly coloured whitish with a pale yellowish periostracum, which is bristly along the posterior keel.

***Gari depressa* (Pennant, 1777)**

Fig. 9B

**References**. von Cosel and Gofas (2019).

**Records**. MAURITANIA - Dakhlet Nouadhibou • Seagrass bed in the Baie de l’Étoile; 21.0224, -17.0062; 16.II.2023; A. Knorrn, M. Sonnewald, S. M. Moctar, and A. Freiwald leg.; push core sampling, BdE-55; IMROP 82 • Submerged ridges; 20.6528, -16.7316; 30.VII.2022; A. Knorrn, S. M. Moctar, F. Krupp, and A. Freiwald leg.; ganchorra haul; BdL-06-1; SMF 372465.

**Identification**. Periostracum a strong brownish to olive colour and persistent on marginal parts of the shell. Interior creamy white to orange and violet with the exterior pattern showing through. Shell oblong to oval in shape and compressed. Surface without conspicuous tent-shaped or zigzag markings. Outside of shell whitish to creamy yellowish with darker commarginal zones and purple, violet, or pale brownish radial rays.

***Gari fervensis* (Gmelin, 1791)**

**References**. von Cosel and Gofas (2019).

**Record**. MAURITANIA - Dakhlet Nouadhibou • Submerged ridges; 20.6528, -16.7316; 30.VII.2022; A. Knorrn, S. M. Moctar, F. Krupp, and A. Freiwald leg.; ganchorra haul; BdL-06-1; IMROP 41, SMF 372459, MAU-248.

**Identification**. Shell more or less elongate and compressed. Posterior slope with ~8 radial riblets that cross the concentric ridges and produce a cancellated pattern. Outside of shell whitish to pale violet, with several darker rays that often show paler mottling on them. Interior pale to dark violet to bright yellow in colour.

***Gari jousseaumeana* Bertin, 1880**

Fig. 6B, 9C

**References**. von Cosel and Gofas (2019).

**Record**. MAURITANIA - Dakhlet Nouadhibou • Baie du Lévrier; 21.0398, -16.9066; 04.XII.2021; S. M. Moctar, and A. Freiwald leg.; grab sample; BdL21-04-2; IMROP 8, SMF366105; MAU-211.

**Identification**. Shell more or less elongate and compressed. Posterior slope with only one conspicuous radial rib crossing the concentric ridges. Outside of shell whitish to pale yellowish or violet, with irregular brownish rays or zigzag pattern. Interior pinkish to violet in colour with intensely coloured parts of the exterior showing through.

***Solen capensis* P. Fischer, 1881**

**References**. von Cosel and Gofas (2019).

**Record**. MAURITANIA - Dakhlet Nouadhibou • Seagrass bed in the Baie de l’Étoile; 21.0277, -17.0035; 25.VII.2022; A. Knorrn, F. Krupp, S. M. Moctar, and A. Freiwald leg.; hand collection; BdE-11-2; SMF 377918.

**Identification**. Shell very elongated, nearly rectangular. Anterior margin obliquely and posterior margin vertically truncated. Surface with irregular growth lines. Shell whitish to yellowish and pale brown, sometimes with darker growth zones. Interior whitish with exterior colouration showing through.

***Callista floridella* (Gray, 1838)**

Fig. 9D

**References**. von Cosel and Gofas (2019).

**Records**. MAURITANIA - Dakhlet Nouadhibou • Kijji island; 19.8227, -16.5940; 20.06.2023, A. Knorrn and A. Freiwald leg.; ganchorra haul; K001; IMROP 130 • Submerged ridges; 20.6528, -16.7316; 30.VII.2022; A. Knorrn, S. M. Moctar, F. Krupp, and A. Freiwald leg.; ganchorra haul; BdL-06-1; SMF 367233.

**Identification**. Shell trigonal ovate in outline. Outer surface with narrow regular concentric grooves posteriorly. Hinge with three irregularly diverging cardinal teeth. Pallial sinus deep and not ascending anteriorly, pointed at anterior end. Outer surface of shell with very irregular or geometric patterns of brown. Interior orange to vermilion with paler to whitish towards the margin.

***Petricola lithophaga* (Retzius, 1788)**

**References**. von Cosel and Gofas (2019).

**Records**. MAURITANIA - Dakhlet Nouadhibou • Rocky island; 21.0252, -17.0070; 19.II.2023; A. Knorrn, S. M.Moctar, M. Sonnewald and A. Freiwald leg.; hand collection; BdE-107; SMF 367207, MAU-452 • Submerged ridges; 20.6878, -16.7131; 04.III.2023; A. Knorrn, S. M. Moctar, M. Sonnewald, and A. Freiwald leg.; ganchorra haul; BdL-86; SMF 367208.

**Identification**. Shell quite irregular, oval, and very inflated. Surface with fine irregular ribs, which are most prominent on the posterior half of the shell and seem to disappear gradually around the middle part of the shell and are missing completely on the anterior half. Shell dirty white and sometimes with a brownish red colouration on the posterio-dorsal slope or the posterior end. Interior white, with the exterior colouration showing through.

***Pitar tumens* (Gmelin, 1791)**

**References**. von Cosel and Gofas (2019).

**Record**. MAURITANIA - Dakhlet Nouadhibou • Submerged ridges; 20.6547, -16.7260; 02.III.2023; A. Knorrn, S. M. Moctar, M. Sonnewald, and A. Freiwald leg.; ganchorra haul; BdL-53; IMROP 163.

**Identification**. Shell rounded ovate and not pointed posteriorly. Shell usually broadly suboval with anterior umbones. Shell rather compressed to moderately inflated. Ventral margin evenly curved, and the lunule is indistinct. Shell surface with very irregular and sometimes prominent growth lines and ridges. Outside of shell creamy white, yellowish, or ochre. Interior white to pale orange.

***Polititapes durus* (Gmelin, 1791)**

**References**. von Cosel and Gofas (2019).

**Records**. MAURITANIA - Dakhlet Nouadhibou • Submerged ridges; 20.6528, -16.7316; 30.VII.2022; A. Knorrn, S. M. Moctar, F. Krupp, and A. Freiwald leg.; ganchorra haul; BdL-06-1; SMF 372466 • Kijji island; 19.8227, -16.5940; 20.VI.2023; A. Knorrn and A. Freiwald leg.; ganchorra haul; K001; IMROP 106.

**Identification**. Outer surface glossy with many concentric ridges and narrow groves. Outside of shell glossy, creamy to pale fawn, often with pale coloured zigzag markings and four interrupted radial bands of dark brown. Interior white to creamy in colour and sometimes pinkish under the beaks.

***Ruditapes decussatus* (Linnaeus, 1758)**

**References**. von Cosel and Gofas (2019).

**Record**. MAURITANIA - Dakhlet Nouadhibou • Seagrass bed in the Baie de l’Étoile; 21.0426, -17.0259; 24.II.2023; A. Knorrn, M. Sonnewald, S. M. Moctar, and A. Freiwald leg.; hand collection; BdE-129; IMROP 73.

**Identification**. Shell solid and inflated, elongate to subquadrate in its outline. Outer surface with fine radial riblets and concentric groves, strongly impressed towards anterior and lateral sides. Pallial sinus deep and reaches the midline of shell or slightly exceeds it. Outside of shell white to pale brownish with various pattern of deep brown. Interior white to yellowish tinted with a purple hue along the ligamental margin.

***Venerupis corrugata* (Gmelin, 1791)**

Fig. 9E

**References**. von Cosel and Gofas (2019).

**Records**. MAURITANIA - Dakhlet Nouadhibou • Submerged ridges; 20.6528, -16.7316; 30.VII.2022; A. Knorrn, S. M. Moctar, F. Krupp, and A. Freiwald leg.; ganchorra haul; BdL-06-1; SMF 367171 • Agadir Sandy beach, Ile d’Arguin; 20.6106, -16.4468; 05.III.2023; M. Sonnewald and A. Knorrn, collected during snorkelling; IdA-09; IMROP 71.

**Identification**. Shell very variable in form but generally rather inflated, oblong and subovate in outline. Surface dull with a very irregular sculpture consisting of irregular, fine, commarginal ridges becoming coarser on the posterio-dorsal part. Outside of shell cream or pale buff to brownish with various brownish patterns. Interior white and often stained with a deep purplish to blue colour on the posterior side.

***Venus casina* Linnaeus, 1758**

**References**. von Cosel and Gofas (2019).

**Records**. MAURITANIA - Dakhlet Nouadhibou • Submerged ridges; 20.6531, -16.7267; 02.III.2023; A. Knorrn, S. M. Moctar, M. Sonnewald, and A. Freiwald leg.; ganchorra haul; BdL-51; SMF 367161 • Baie du Lévrier; 20.8705, -17.0377; 02.III.2023; A. Knorrn, M. Sonnewald, S. M. Moctar, and A. Freiwald leg.; grab sample; BdL-42-2; IMROP 62.

**Identification**. Shell variable in shape but mostly suboval to circular and more or less inflated. Surface of shell with thin, irregular, commarginal lamellae. The prominent lamellae are often slightly bent towards the dorsal side. Outside of shell of shell pale yellowish to fawn-coloured with brownish to purplish rays, dots, and zigzag markings. Interior white, sometimes with a pale pinkish hue.

**Remarks**. Similar to *Venus verrucosa*, but without diverging rows of warty tubercles on the anterior and posterior parts of the shell.

***Venus verrucosa* Linnaeus, 1758**

Fig. 9F

**References**. von Cosel and Gofas (2019).

**Records**. MAURITANIA - Dakhlet Nouadhibou • Seagrass bed in the Baie de l’Étoile; 21.0248, -17.0071; 17.II.2023; A. Knorrn, M. Sonnewald, S. M. Moctar, and A. Freiwald leg.; hand collection; BdE-63; IMROP 58, SMF 367157 • Maerl bed in the Baie de l’Étoile; 21.0207, -17.0056; 16.II.2023; A. Knorrn, M. Sonnewald, S. M. Moctar, and A. Freiwald leg.; hand collection; BdE-45; IMROP 59 • Submerged ridges; 20.6528, -16.7316; 30.VII.2022; A. Knorrn, S. M. Moctar, F. Krupp, and A. Freiwald leg.; ganchorra haul; BdL-06-1; IMROP 44, SMF 372462.

**Identification**. Escutcheon with transverse brown markings on the left valve. Interior white and sometimes posteriorly maculated with brown. Shell rounded ovate with a pronounced escutcheon on the left valve. Outer surface with lamellose and prominent concentric ridges forming diverging rows of warty tubercles. Outside of shell dirty white to brownish with pinkish to brown markings.

**Remarks**. Similar to *Venus casina*, but with prominent diverging rows of warty tubercles on the anterior and posterior parts of the shell.

***Sepia officinalis* Linnaeus, 1758**

Fig. 10A

**Figure 10.**
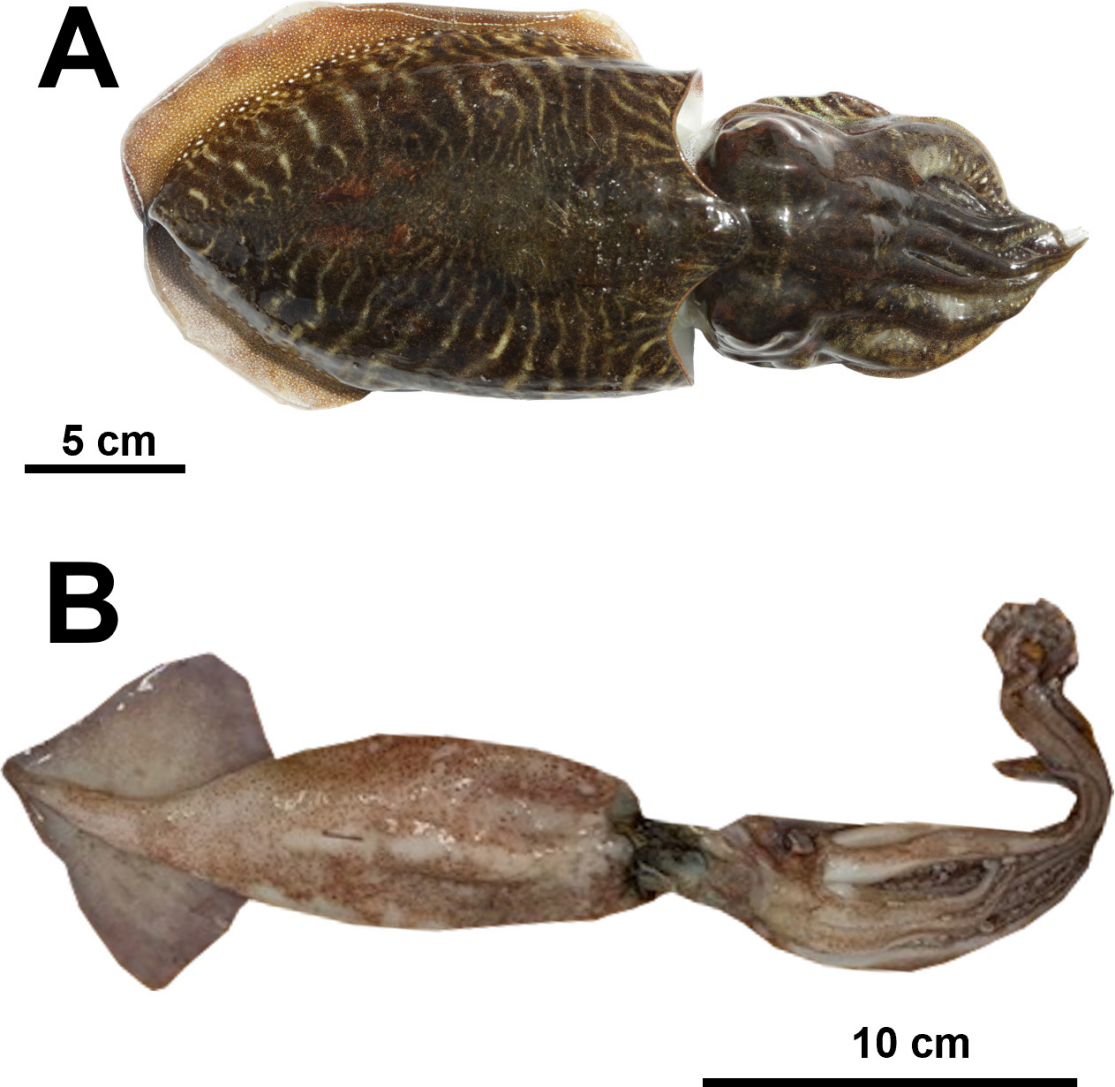
Cephalopods from coastal habitats and artisanal fish markets in Mauritania. **A**. *Sepia officinalis*; **B**. *Todarodes sagittatus*. © Kristina Hopf: A, © Mamadou Dia: B.

**References**. Hayward and Ryland (2017).

**Record**. MAURITANIA - Dakhlet Nouadhibou • Seagrass bed in the Baie de l’Étoile; 21.01201, -17.0133; 25.VII.2022; F. Krupp and A. Freiwald leg.; Beach seine; BdE-08; IMROP 47, MAU-459.

**Identification**. Body broadly oval and rather flattened. Dorsal and anterior part of the mantle form a blunt and rounded lobe. Arms with four rows of suckers. Fins extend along the entire length of the body. Body colour variable but mostly pale brown. Head with several white spots. Dorsal side of the mantle with a zebra-striped pattern during the breeding season.

***Todarodes sagittatus* (Lamarck, 1798)**

Fig. 10B

**References**. Carpenter and De Angelis (2016a).

**Record**. MAURITANIA - Nouakchott Nord • Nouakchott market; 18.1033, -16.0263; VII.2021; M. Dia and A. Niang leg.; fish market survey (tissue sample).

**Identification**. Cartilaginous funnel with a distinct inverted T-shaped groove. Arms with two rows of suckers. Arm suckers with one enlarged central tooth and 8–10 regular teeth and no small alternating teeth. Tentacular club with four rows of suckers with the median ones being larger. The tentacular club extends along the stalk and occupies ~75–83% of the arm length.

#### Brachiopoda

***Lingula parva* Smith, 1872**

Fig. 11

**Figure 11.**
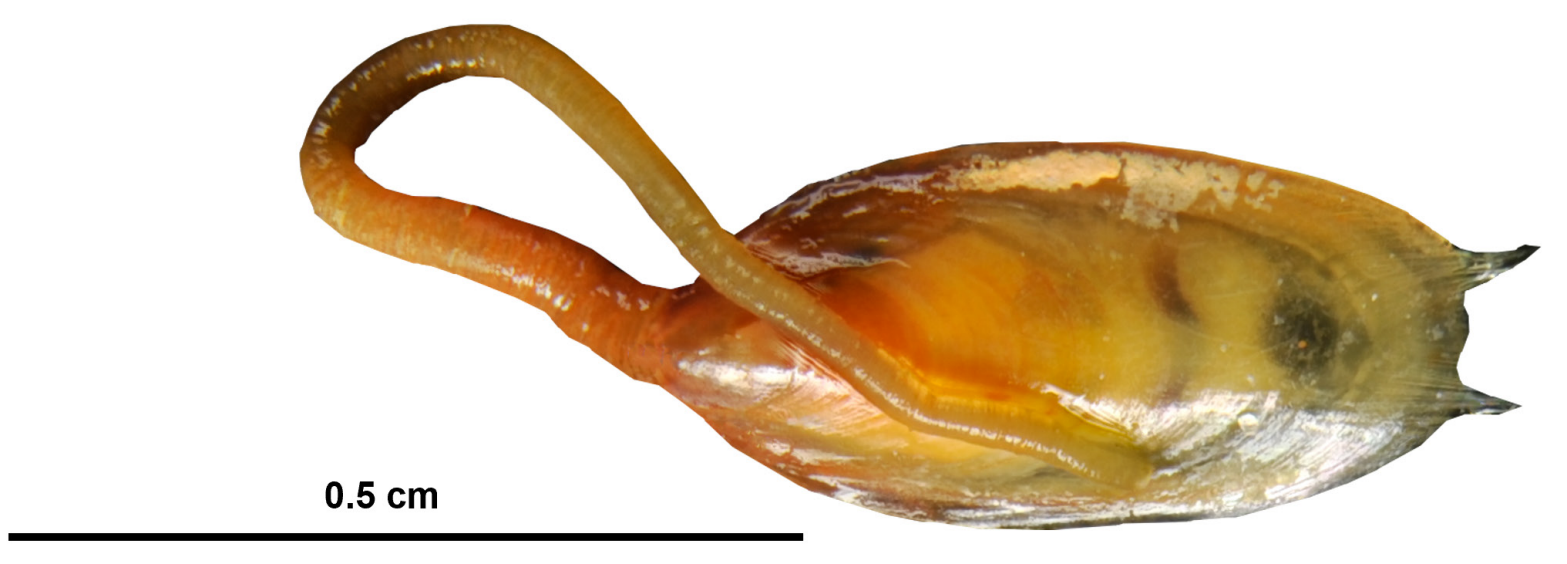
A living marine brachiopod (*Lingula parva*) collected twice at coastal habitats in Mauritania © Nicol Mahnken.

**References**. Emig and Le Loeuff (1978).

**Records**. MAURITANIA - Dakhlet Nouadhibou • Baie de Archimede; 21.0398, -16.9066; 04.XII.2021; A. Freiwald leg.; Grab sample; BDL21-4-2; SMF 866 • Kijji island; 19.8227, -16.5940; 20.VI.2023; A. Knorrn and A. Freiwald leg.; ganchorra haul; K001; SMF 865.

**Identification**. Shell elongated and oval in shape, weakly transparent, and calcified with several definite muscular insertions. Ventral valve extends beyond the dorsal valve. Shell and body pale brownish to greyish brown and to copper in colour.

#### Echinodermata

***Asterina gibbosa* (Pennant, 1777)**

Fig. 12A

**Figure 12.**
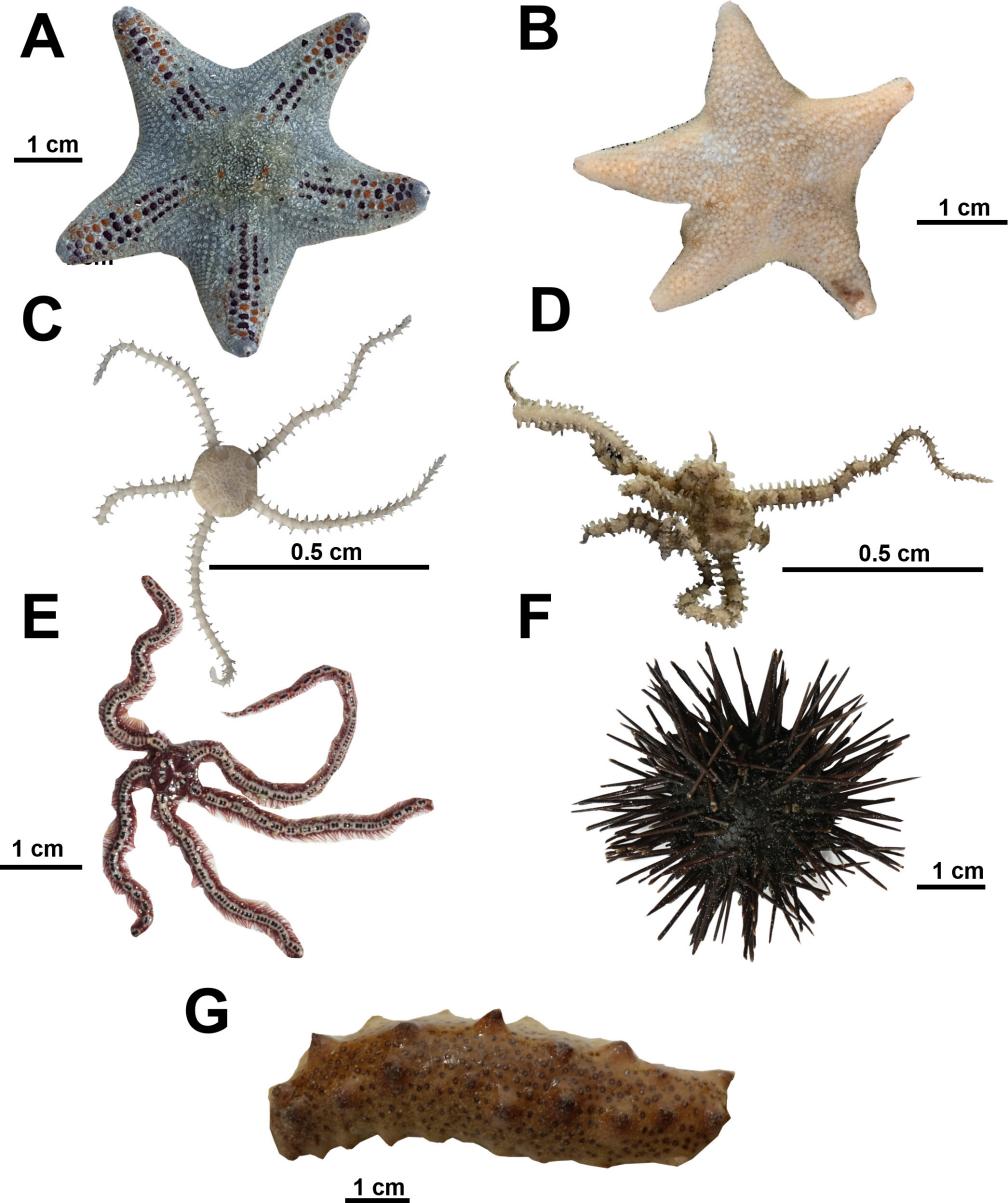
Echinoderms from coastal habitats in northern Mauritania. **A**. *Asterina gibbosa*; **B**. *Asterina stellifera*; **C**. *Amphipholis squamata*; **D**. *Ophiactis lymani*; **E**. *Ophiothrix cotteaui*; **F**. *Arbacia lixula*; **G**. *Holothuria arguinensis*. © Nicol Mahnken: A, B, C, D, © Kristina Hopf: E, F, G.

**References**. Southward and Campell (2006).

**Record**. MAURITANIA - Dakhlet Nouadhibou • Maerl bed in the Baie de l’Étoile; 21.0184, -17.0030; 20.II.2023; A. Knorrn, M. Sonnewald, S. M. Moctar, and A. Freiwald leg.; hand collection; BdE-119; SMF 6961.

**Identification**. Body roughly pentagonal in shape with very short arms. Upper side slightly swollen with sharp edges. Lower side flat. Spines on the upper side arranged in groups of 4–8. Spines of the lower side arranged in groups of two or three. Uniformly to mottled pale brown to grey and olive green in colour.

**Remarks**: Smaller individuals can have a resemblance to *A. stellifera* but differ in the less pointed arms and irregular arrangement of abactinal plates.

***Asterina stellifera* (Möbius, 1859)**

Fig. 12B

**References**. Clark and Downey (1992).

**Record**. MAURITANIA - Dakhlet Nouadhibou • Pelican island; 20.7111, -16.6880; 03.III.2023; A. Knorrn, M. Sonnewald, S. M. Moctar, and A. Freiwald leg.; hand collection; BdL-74; IMROP1, SMF 6960, MAU-359.

**Identification**. Short-rayed sea star with evenly tapering, almost pointed, rays. Abactinal skeleton with a reinforced pentagon on the disc formed by plates linking the five primary radials. Other primary plates mostly elongated and lunate in shape. Greyish to olive in colouration with occasional orange or reddish spots. Body thick and laterally sloping.

**Remarks**: Smaller individuals have a resemblance to *A. gibbosa* but differ externally in more pointed arms and a more regular arrangement of abactinal plates.

***Amphipholis squamata* (Delle Chiaje, 1828)**

Fig. 12C

**References**. Hayward and Ryland (2017).

**Record**. MAURITANIA - Dakhlet Nouadhibou • Maerl bed in the Baie de l’Étoile; 21.0206, -17.0045; 16.II.2023; A. Knorrn, M. Sonnewald, S. M. Moctar, and A. Freiwald leg.; hand collection; BdE-56; SMF 6968. • Kijji island; 19.8249, -16.5954; 20.VI.2023, A. Knorrn leg.; hand collection while diving; K003; SMF 62514.

**Identification**. Dorsal disc covered by small scales. Radial shields equal to one third of the dorsal disc radius and joined for almost the entire length. Dorsal arm plates rounded, triangular, and broader than long at the proximal apex. Three or four proximal arm spines present. Arms and disk greyish to pale brown in colour.

***Ophiactis lymani* Ljungman, 1872**

Fig. 12D

**References**. Borges et al. (2002).

**First records**. MAURITANIA - Dakhlet Nouadhibou • Rocky island; 21.0252, -17.0070; 19.II.2022; A. Knorrn, F. Krupp, S. M. Moctar, and A. Freiwald leg.; hand collection; BdE-107; IMROP 3, SMF 6965, MAU-476 • Maerl bed in the Baie de l’Étoile; 21.0248, -17.0071; 17.II.2023; A. Knorrn, M. Sonnewald, S. M. Moctar, and A. Freiwald leg.; hand collection; BdE-63; SMF 6967, MAU-478.

**Identification**. Disc covered in large irregular scales. Radial shields twice as long as wide. Sparsely distributed spines along the marginal region of the disc. Behind the radial shield there are two blunt spines. Two oral papillae on each side of the mandible and a pair of irregular infradentals at the apex. Dorsal brachial plate flabelliform and ventrally rounded on the posterior margin. The five arms and the discs are creamy white to dark grey with a dark brownish marbled pattern.

***Ophiothrix cotteaui* (de Loriol, 1900)**

Fig. 12E

**References**. de Loriol (1900).

**Record**. MAURITANIA - Dakhlet Nouadhibou • Submerged ridges; 20.6562, - 16.7199; 04.II.2024; A. Knorrn, S. M. Moctar, M. Sonnewald, and A. Freiwald leg.; ganchorra haul; BdL-122; SMF 6964.

**Identification**. Central disc covered in fine grains instead of spines. Radial plates very large, absolutely naked, and shaped like an inequilateral triangle with the short side more or less parallel to the rim. Five arm spines at the proximal end and four arm spines at the distal end of the arms. Body and arms coloured in a dark red to dark violet with a whitish marbled pattern on the dorsal side of the disc.

***Arbacia lixula* (Linnaeus, 1758)**

Fig. 12F

**References**. Riedl (1983).

**Record**. MAURITANIA - Inchiri • Sandy beach at Blaouakh; 18.5187, -16.0750; 23.I.2024; A. Knorrn, K. Pfennings, and K. Hopf leg.; hand collection; B-02; IMROP 2, SMF 6962.

**Identification**. Aboral ambulacral plate with three pairs of pores and deep gill incisions. Aboral suction feet without suction disc. Black body with black spines. Spines aproximately as long as the body diameter.

**Holothuria (Roweothuria) arguinensis Koehler & Vaney, 1906**

Fig. 12G

**References**. González-Wangüemert and Borrero-Pérez (2012).

**Record**. MAURITANIA - Inchiri • Sandy beach at Blaouakh; 18.5187, -16.0750; 24.II.2023; A. Knorrn, K. Pfennings, and K. Hopf leg.; hand collection; B-02; SMF 6963.

**Identification**. Body cylindrical with large papillae or warts on the dorsal surface. Warts or papillae blackish brown to whitish and arranged in six longitudinal rows. Body with a uniformly yellowish colouration with a browner dorsal side. Whitish dots distributed along the body with punctations that match the retracted pedicels, which are pale brown.

Additional species that could not be identified to species level or for which no exact location could be determined are listed in Table 10. Further research of specialised taxonomists is urgently needed to identify these species.

**Table 10.** List of observed species with no exact location (NEL) and/or no species identification (NSI). Location abbreviations provided in Table 1.

Species	Location	Status	Remarks
** Cnidaria **			
*Filigorgia* sp.	SRF, BB	NSI	Different morphotypes of this genus were found along the sandy beaches of Mauritania and within the submerged ridge fields. Due to an unclear taxonomy, no species identification could be made.
Hydrozoa indet.	RI, OP, PI, SRF,	NSI	Various morphologically different hydrozoan species were observed at different habitats, but no exact species identification could be made.
** Crustacea **			
Tanaidacea indet.	S-BdE	NSI	Due to the lack of time and experts, no species identification has yet been made.
Isopoda indet.	RI, OP, PI	NSI	Only one species (*Sphaeroma serratum*) was morphologically identified. Additional species are likely to be found.
Amphipoda indet.	RI, OP, PI, SRF, CP	NSI	Due to the lack of time no species identification has yet been made.
*Calappa rubroguttata* Herklots, 1851	BB	NEL	Two dead individuals were found within abandoned fishing gear.
** Mollusca **			
*Pteria atlantica* (Lamarck, 1819)	CP, BB	NEL	Found attached to abandoned fishing nets.
*Cardium costatum* Linnaeus, 1758	BB	NEL	Only the shells were found washed up to the beach.
** Tunicata **			
*Microcosmus* Heller, 1877 sp.	BB, RI, SRF	NSI, NEL	Several observations were made but no specific species identification could be made. Additional individuals were found washed up on the beach at BB.
cf. *Stolonica socialis* Hartmeyer, 1903	SRF	NSI	Great abundances along the SRF, but no clear taxonomic identification for approval.
** Porifera **			
*Cinachyrella Wilson, 1925*	OP, RI, SRF	NSI	Great abundances of this sponge species occurred along the Mauritanian coast. No exact species identification could be made.
Porifera indet.	SRF	NSI	Various morphologically different sponge species were observed along the SRF, but no exact species identification could be made.
** Polychaeta **			
Polychaeta indet.	S-BdE, BdL, SRF, OP, RI,	NSI	Several polychaetes smaller than 1 cm were found. No precise species identification could be made, additional DNA barcoding and phylogenetic analyses are required.
** Plathelminthes **			
Plathelminthes indet.	RI, OP	NSI	Different specimens were collected, but no taxonomic identification could be made
** Bryozoa **			
Bryozoa indet.	S-BdE, SRF, OP	NSI	Various morphologically different bryozoan species were observed, but no exact species identification could be made.

## Discussion

Within this study, 103 different living macrozoobenthic species from the northern Mauritanian coastal habitats were observed (Cnidaria: 2, Crustacea: 33, Polyplacophora: 2, Gastropoda: 28, Bivalvia: 28, Cephalopoda: 2, Brachiopoda: 1, Echinodermata: 7). A total of 14 species were not known from Mauritania and are therefore treated as new records. This again, highlights the urgent need for biodiversity and taxonomic research along the Mauritanian marine habitats as outlined in Knorrn et al. (2025b). Some animal groups showed a conspicuously high diversity of species in correlation with certain habitats. For example, most of the observed bivalve species were found at the submerged ridge field habitats. Like the habitat-forming octocorals and ascidians of the submerged ridge fields, the bivalves also exhibit a filter-feeding lifestyle. The current conditions along this habitat, potentially influenced by strong tidal currents and a rich supply of nutrients, may favour animal groups that primarily filter-feed. Gastropods and crustaceans were evenly distributed between seagrass beds and hard substrate habitats. Both habitats are intertidal and offer shelter and hiding places. In particular, seagrass beds may become especially important and more frequent along the Mauritanian coast in the future. With rising sea levels, the erosion of coastal sand will increase, which could lead to the formation of new shallow-water lagoons and sebkhas due to dune breaches, similar to those observed in the Bellaat lagoon during August 2013 (Trégarot et al. 2020). However, with ongoing sea-level rise, many coastal areas around Mauritania, including coastal cities and villages like the capital city Nouakchott, are at great risk of flooding in the upcoming years (Senhoury et al. 2016; Knorrn et al. 2025b).

Due to the unavailability of precise sampling locations for specimens (or body parts) ashore, entangled in abandoned fishing gear, or not yet fully identified to species level, some observations were excluded from the checklist and are mentioned in Table 10. These unidentified specimens highlight the actual lack of literature and taxonomic work in the area. Further investigations and newly built taxonomic expertise are required to identify these to expand our knowledge of the Mauritanian marine fauna. Considering this, the list highlights that much remains to be discovered in Mauritanian waters, and there are still many significant knowledge gaps, especially for non-commercial and smaller macrozoobenthic species. Studies like these are of particular importance as a basis for studies on local food webs, where many more than just commercially used species have to be considered. Scientific collections play a particularly important role in overcoming this knowledge gap in Mauritania, as they serve as the basis for further scientific work. By making specimens identified to species level available to other scientists, these vouchers can be used for comparative work. Additionally, scientific collections serve as valuable reference points for future research, offering information on past biodiversity that can be compared with present or future states (Carter et al. 2001; Sanders et al. 2023). To address this need, IMROP and SRI are working together to establish and continuously expand the scientific reference collections of the Mauritanian marine fauna in both Frankfurt am Main (Germany) and Cansado (Mauritania). These scientific collections currently consist of specimens mentioned herein, as well as additional fish vouchers mentioned in a previous study (Knorrn et al. 2025a). Still, the listed occurrences herein are incomplete and the given locations are examples of typical habitats of the respective species. The connection between metadata and voucher specimens follows the extended specimen approach (Lendemer et al. 2020) and allows a broader use of the collected data.

This checklist presents our current state of knowledge of the invertebrate macrofauna from north Mauritanian coastal habitats. It supports future ecosystem-based management planning by providing a baseline of frequently traded but also lesser-known invertebrates from these habitats and artisanal landing sites. Additionally, the most outstanding morphological characteristics of each species are described in detail and to aid in identification for future work. Further investigations are needed to provide a more detailed and comprehensive list of the marine fauna occurring in Mauritanian waters, especially focusing on smaller invertebrates as well as cnidarians, tunicates, and sponges. Checklists like these in combination with a reference collection are very valuable sources for future biodiversity assessments, especially in the context of anthropogenically induced environmental change, enhanced by increased exploitation of fish resources in our oceans.

## Additional information

### Use of AI

No use of AI was reported.

### Author ORCIDs

Alexander H. Knorrn https://orcid.org/0009-0009-3254-3505

Moritz Sonnewald https://orcid.org/0000-0003-3042-8107

Sidi Mohamed M. Moctar https://orcid.org/0009-0005-6648-1809

Mamadou Dia https://orcid.org/0000-0003-0134-1547

Ely Beiobou https://orcid.org/0009-0005-0856-6249

André Freiwald https://orcid.org/0000-0002-2335-4042

### Data availability

The generates main data of this study are available in the main text. Additionally, the occurrence data will be included within GBIF (https://www.gbif.org/) database and the mentioned DNA-Barcodes will be available on the BOLD-Systems DNA-Barcode database as part of the Mauritanian marine life barcode library (MAU) project.
